# A K-17 serotype specific Klebsiella phage JKP2 with biofilm reduction potential

**DOI:** 10.1016/j.virusres.2023.199107

**Published:** 2023-04-02

**Authors:** Muhammad Asif, Iqbal Ahmad Alvi, Muhammad Waqas, Abdul Basit, Faiz Ahmed Raza, Shafiq-ur Rehman

**Affiliations:** a Institute of Microbiology and Molecular Genetics, University of the Punjab, Lahore, Pakistan; b Department of Pathology, King Edward Medical University, Lahore, Pakistan; c Department of Microbiology, Hazara University, Mansehra, Pakistan; d Health Research Institute, National Institute of Health, Research Centre, King Edward Medical University, Lahore, Pakistan

**Keywords:** Antimicrobial resistance, *Klebsiella pneumoniae*, Bacteriophage, Phage therapy, Biofilm, Whole genome, Klebsiella phage, serotype

## Abstract

*Klebsiella pneumoniae* is an opportunistic pathogen responsible for nearly one-third of all Gram-negative infections. Increasing antibiotic resistance has pushed scientists to look for alternative therapeutics. Bacteriophages have emerged as one of the promising alternatives. In the current study, the Klebsiella phage JKP2 was isolated from a sewage sample and characterized against the K-17 serotype of *K. pneumoniae*. It produced bulls-eye-shaped clear plaques and has a latent period of 45 min with a burst size of 70 pfu/cell. It remained stable at tested pH (5 to 10) and temperatures (37 to 60 °C). Its optimum temperature for long-term storage is 4 °C and -80 °C. The JKP2 showed its infectivity against the *K. pneumoniae* K-17 serotype only. It controlled planktonic cells of *K. pneumoniae* 12 h post-incubation. At MOI-1, it efficiently eliminated 98% of 24 and 96% of 48-hour-old biofilm and 86% and 82% of mature biofilm of day 3 and 4, respectively. The JKP2 has an icosahedral capsid of 54 ± 0.5 nm with a short, non-contractile tail, measuring 12 ± 0.2 nm. It possesses a double-stranded DNA genome of 43.2 kbp with 54.1% GC content and encodes 54 proteins, including 29 with known functions and 25 with unknown functions. JKP2 was classified as *Drulisvirus* within the *Autographiviridae* family. It uses a T7-like direct terminal repeat strategy for genome packaging. JKP2 can be applied safely for therapeutic purposes as it does not encode an integrase or repressor genes, antibiotic resistance genes, bacterial virulence factors, and mycotoxins.

## Highlights

• JKP2 is a first isolated phage against *Klebsiella* K-17 capsular serotype.
• It produced bulls eye shaped clear lytic plaques due to tail associated depolymerase.
• JKP2 remained stable at tested pH (5 to 10) and temperatures (37 to 60 °C).
• JKP2 showed *K. pneumoniae* biofilm reduction potential.
• Based on whole genome sequence analysis it belong to unclassified *Drulisvirus* within the *Autographiviridae* family.

## 1 Introduction

*Klebsiella pneumoniae* is an opportunistic bacterial pathogen that is a member of the *Enterobacteriaceae* family. It is an important colonizer of the human mucosal surfaces without causing any pathology. However, it can cause severe life-threatening diseases when disseminated to other tissues (Paczosa and Mecsas, 2016). It accounts for a substantial number of community-acquired and hospital-acquired infections worldwide, particularly in the elderly, neonates, and immunocompromised individuals. It is responsible for approximately one-third of all Gram-negative infections, including pneumonia, urinary tract infections, cystitis, septicemia, endocarditis, and surgical wound infections. It is also implicated in the development of necrotizing ventilator-associated pneumonia (VAP), hospital-acquired pneumonia (HAP), pyogenic liver abscesses, meningitis, and endogenous endophthalmitis (Effah et al., 2020).

The emergence and rapid global spread of multi-drug resistant (MDR) and extensively drug-resistant (XDR) organisms, particularly microbes from the *Enterobacteriaceae* family, pose a serious threat to human health. Such antibiotic-resistant organisms cause higher morbidity and mortality, with extended hospitalization and higher treatment costs than other organisms. Due to its increasing antibiotic resistance acquisition, *K. pneumoniae* is now considered as a serious public health threat. It is classified as an ESKAPE organism, a highly virulent and antibiotic-resistant group of pathogens (Navon-Venezia et al., 2017).

*K. pneumoniae* is renowned for its propensity to create biofilms consisting of a thick coating of extra polymeric substances that encourages bacterial attachment to living or non-living surfaces, preventing antibiotics from penetrating and limiting their efficacy (Nirwati et al., 2019). Antibiotic resistance is ten to one thousand-fold greater in mature bacterial biofilms than in planktonic bacterial cells. Furthermore, bacteria in biofilms are resistant to phagocytosis, making them extremely difficult to eradicate (Zheng et al., 2018).

Antibiotics are becoming less effective day by day, and very few new drugs have been developed in the last two decades despite massive efforts. Therefore, there is a need to explore new avenues to curtail these drug-resistant bacterial infections. Among many other explored alternates, bacteriophages have emerged as promising candidates. They have several advantages over antibiotics, especially host specificity, auto increase in number after multiplication in host at infection site, self-elimination after host eradication, and availability of a vast repertoire of phages (Zurabov and Zhilenkov, 2021).

Phages are potential candidate as an alternative therapeutic option against *Klebsiella* infections. In past decade anti-Klebsiella phages have been employed to treat liver abscesses, wound infections, bacteremia, and pneumonia in animal model of infection (Chhibber et al., 2008; Kumari et al., 2009). There are several case-studies that employed anti-Klebsiella phage as last resort treatment in drug-resistant infections in humans. Kuipers et al. (2019) published a case report on the successful treatment of a 58-year-old renal transplant patient with chronic UTI by ESBL-Kp. In another study, a 62-year-old diabetic patient with a prolonged history of repeated prosthetic joint infections with *K. pneumoniae* was treated with phages. Eskenazi et al. (2022) reported a patient with a polytraumatic fracture infected with pan-drug-resistant *K. pneumoniae*. The infection was treated with a combination of various antibiotics and pre-adapted bacteriophage. Moreover, some countries have started their own compassionate phage therapy centers to treat recalcitrant bacterial infections (Żaczek et al., 2020). There is a great diversity among pathogenic bacteria and phages as well, therefore, they are developing their own phage banks against variety of bacteria. Phages are the potential alternative treatment options against XDR bacterial infections (Serwer et al., 2021).

This study was aimed at isolating the new bacteriophage. The new bacteriophages must be characterized for parameters necessary for therapeutic application, such as thermal and heat stability, storage temperature, latent period, burst size, planktonic and biofilm eradication ability, and genetic characterization. This study focused on isolating and characterizing bacteriophages against MDR *K. pneumoniae*, biofilm eradication, and complete genome analysis.

## 2 Materials and methods

### 2.1 Ethical considerations

This study did not involve any direct interaction with human or animal subjects; therefore, ethical approval was not sought. All pathogens were handled in a BSL-2 level laboratory in biosafety cabinet type 2.

### 2.2 Isolation and characterization of bacterial strains

Bacterial strains were isolated by standard culturing of clinical samples referred to the Department of Pathology, King Edward Medical University, Lahore. The isolated organisms were identified by API 20E and 20NE, followed by 16S rDNA amplification and sequencing. Colony-PCR was carried out with universal primers, 27F (5′-AGAGTTTGATCCTGGCTCAG-3′) and 1492R (5′-GGTTACCTTGTTACGACTT-3′) for amplification of 16S rDNA followed by PCR purification and Sanger sequencing. Homology and phylogenetic analysis were carried out for these sequences and submitted to NCBI. The stocks of samples were stored in 25% glycerol at −80°C for further experiments. Running cultures were maintained in Luria*–*Bertani (LB) broth and LB agar. The antibiotic susceptibility profile was assessed by the Kirby-Bauer disk diffusion method according to the CLSI guidelines 2019. The sample source and antibiotic resistance profile are enlisted in Table S1.

### 2.3 Genetic analysis of isolated strains

DNA extraction of bacterial strains was carried out using GeneJET Genomic DNA Purification Kit as per the manufacturer's instructions (Thermo Scientific™, Catalog# K0722). The purity of DNA was assessed by agarose gel electrophoresis and nano-drop spectrophotometer. Whole-genome sequencing was carried out by Nextera library preparation followed by data generation through Hiseq 200 PE. . The reads were analyzed for quality, trimmed and them assembled with SPAdes. *Klebsiella* serotyping for K-1, K-2, K-5, K-20, K-54, and KL-5 was carried out by PCR as reported previously (Turton et al., 2010). Serotype prediction of some selected strains was made by analyzing the whole genome through Pathogenwatch that uses the Kaptive tool (https://cgps.gitbook.io/pathogenwatch/) to predict K and O locus. Whole-genome multi-locus sequence typing (MLST) was carried out with MLST-2.0 Server (https://cge.cbs.dtu.dk/services/MLST/). The presence of virulence factors and antibiotic-resistant genes were analyzed through the virulence factor database (VFDB) (http://www.mgc.ac.cn/VFs/) and ResFinder (https://cge.cbs.dtu.dk/services/ResFinder/), respectively.

### 2.4 Isolation, purification, and enumeration of phages

*K. pneumoniae* strain 8890 (Kp-8890) was used as the host strain to isolate phages from Jinnah Hospital Lahore sewage samples. Spot assay was used to check the presence of bacteriophages, and the double-layer agar technique was used for plaque assay and titer calculation, as reported previously *(**Asif et al., 2021**)*. An average phage titer was calculated after three independent double-layer agar experiments. The purified phages were stored in SM buffer at 4 °C for further characterization experiments and at −20 and −80 °C with 25% glycerol for extended storage*.*

### 2.5 Determination of optimal multiplicity of infection (MOI)

The optimal phage-host bacterial ratio (MOI) was determined that yields maximum titer. The host strain (Kp-8890) was grown to 10^12^ CFUs/mL, and JKP2 phage lysate was added at MOI of 10, 1, 0.1, 0.01, and 0.001. The mixture was incubated for 24-hours at 37°C with continuous shaking at 150 rpm, followed by titer determination through the double-layer agar technique.

### 2.6 Host range assessment

The intraspecies host range of JKP2 was assessed by spot tests against 11 strains of *K. pneumonia*e (Table 1 and S1). Interspecies host range was assessed against already characterized non-ATCC in-house clinical strains kindly provided by the Microbiology Laboratory, Mayo-Hospital, Lahore (Data not shown). A total of 5 *Escherichia coli,* 3 *Enterobacter*, 5 *Pseudomonas*, and 5 *Staphylococcus aureus* isolates were tested. The efficiency of plating (EOP) was determined against spot positive strains, and phages with an EOP of > 0.1 were classified as highly virulent.

**Table 1:** Activity of JKP2 phage against selected *Klebsiella* strains.

*Klebsiella* strain	*Klebsiella* serotype	Sequence type by MLST	Spot assay*	Titer (pfu/mL)	EOP
Kp-8890	K-17	ST-870	+	2.35 × 10^9^	1
Kp-9819	K-17	ST-870	+	2.0 × 10^9^	0.85
K-36	K-17	ST-395	+	2.15 × 10^9^	0.91
Kp-37	K-17	ST-870	+	2.11 × 10^9^	0.89
Kp-11	K-158	ST-397	–	–	
Kp-32	K-20	Unknown	–	–	
Kp-Eb2	K-1	Unknown	–	–	
Kp-KPU	K-112	ST-15	–	–	
Kp-25	Unknown	Unknown	+	2.95 × 10^9^	1.25
Kp-8968	Unknown	Unknown	–	–	
Kp-8973	Unknown	Unknown	–	–	

**Abbreviations: EOP** = efficiency of plating, **pfu** = plaque-forming unit.

⁎ + indicate clear lytic spot, and – indicate absence of any lytic spot.

### 2.7 Growth reduction assessment against planktonic cells

The growth inhibition efficacy of JKP2 was assessed against *K. pneumoniae* planktonic cells, a sterile flask with a known titer of bacteria (2.4 × 10^8^ CFUs/mL) in LB broth was incubated with appropriate volume of phage lysate of known titer (2.35 × 10^9^ pfu/mL) at a MOI 0.1 and 1. The flasks with bacteria only and one with sterile LB broth were taken as positive and negative controls, respectively. The flasks were incubated at 37 °C with continuous shaking at 150 rpm for 12 h, and OD_600_ was taken at 2-hour intervals.

### 2.8 Time-Kill curve

The time-kill analysis was performed as reported previously (Pereira et al., 2016) with some modifications. Briefly, the Kp-8890 strain was grown to 1 × 10^6^ CFUs/mL and the cells were harvested by centrifugation followed by suspension in 100 mL of LB broth. An appropriate volume of phage lysate was added to obtain MOI of 0.1 and 1. A positive control containing bacteria only and a negative control containing LB broth were also set up alongside the experiment. The flasks were incubated at 37°C with shaking at 150 rpm. One milliliter aliquots were withdrawn at 0, 2, 4, 6, 8, 10, 12, 16, 20 and 24 h of incubation from tests and controls and bacterial titer was determined. Decrease in cell counts in tests was compared with the zero-time point and untreated positive control to determine the killing efficacy of phage. The experiment was carried out in duplicate and two independent experiments were conducted to calculate the average kill efficiency.

### 2.9 Determination of bacteriophage-insensitive mutants frequency

The frequency of bacteriophage-insensitive mutants (BIMs) emergence was determined by already reported method (García et al. (2007) with some modifications. Briefly, five colonies of Kp-8890 were grown overnight at 37°C in 50 mL LB broth. The individual bacterial cultures were diluted to adjust bacterial count up to 10^8^ CFU/mL. Cells from 1-mL of culture were collected and appropriate volume of phage suspension was added to obtain MOI of 1 and 0.1 in a total 10 mL of LB broth. The mixture was incubated at 37°C for 10 min. Serial dilutions of the mixture were plated on LB agar followed by incubation at 37°C for 48-hours. Resulting colonies were counted, and the BIM frequency was determined by dividing the surviving viable counts by the initial viable counts. Average BIMs from five colonies were calculated. A positive control containing bacteria only and negative control containing phages only in LB broth was also set up alongside. All the experiments were performed in duplicate and two independent experiments were conducted.

### 2.10 Biofilm formation kinetics by host bacterium

The biofilm formation potential of Kp-8890 in polystyrene microtiter plates was studied by the crystal violet (CV) dye-binding and viable cell count method as reported previously (Haney et al., 2021; Tabassum et al., 2018b). The biofilm formation was studied for six consecutive days. The colony counts of each day and OD_595_ were plotted against time to know the maturation stage of biofilm. The colony counts obtained on each day will be used for approximate calculation of MOI for biofilm treatment.

### 2.11 Assessment of JKP2 to remove established biofilm in a microtiter plate

Treatment protocol was designed according to guidelines published by Abedon et al. (2021). Established biofilms of different ages (1–4 days) were washed three times with physiological saline, and 100 µL of fresh LB broth was added to each well. For approximate MOI calculation, cell counts from preformed biofilm on 24, 48, 72 and 96-hours corresponding to 2.05 × 10^4^, 1.99 × 10^6^, 2.11 × 10^9^ and 2.30 × 10^9^ were used respectively. JKP2 phage lysate stock titer (3.71 × 10^9^ pfu/mL) suspended in SM-buffer was diluted if required to obtain calculated volume of JKP2 to obtain MOI-1 and 0.1 in accordance to bacterial counts. The total volume of microwell was adjusted up to 200 µL. Two wells with only biofilm growth and two with sterile broth were used as positive and negative control respectively. Sterile SM-buffer was added to positive control (mock treatment control) in place of phage lysate. The viable cell count was determined at zero-time point and after 6, 12 and 24-hours of treatment for both the treated and untreated control groups. Log reduction in CFUs/mL was calculated and compared to zero-time point and untreated biofilm growth control. Log reduction in CFUs/mL was calculated by the following formula:

Log reduction = log10(T/Z), whereas *T* = bacterial CFUs/mL in treated samples and *Z* = bacterial CFUs/mL at zero-time points and/or untreated biofilm growth control. Percent reduction in CFUs/mL was also calculated by following formula:

Percentage reduction = (1- T/U) × 100, Whereas *T* = bacterial CFUs/mL in treated samples and U = bacterial CFUs/mL in untreated samples. To obtain microscopic images, biofilm was CV stained as reported previously by Haney et al. (2021) and imaged via inverted microscope at 20X magnification (IRMECO GmbH Germany, Model IM-200).

### 2.12 Stability of JKP2 at varying pH and temperatures

JKP2 phage (2.35 × 10^9^ pfu/mL) was subjected to heat and pH treatment for one and two hours, followed by phage titer determination. A pH range of 3–10 and varying temperatures (37, 45, 60, and 80 °C) were used. To assess the long-term storage stability, JKP2 was stored at varying temperatures (25, 4, −20, and −80 °C) for one, six, and twelve months, followed by titer determination. One-way ANOVA was applied to compare the groups. Tukey's test was used for multiple comparison.

### 2.13 Determination of latent time and burst size

Exponentially grown bacteria cells corresponding to 1 × 10^8^ CFU/mL were collected by centrifugation at 2000 X g for 5 min. The pellet was re-suspended in 500 µL of LB broth. JKP2 phage lysate was added to it at an MOI 0.1, followed by incubation at 37 °C for 1 min. The mixture was centrifuged at 10,000 X g for 30 s to remove unadsorbed bacteriophages. The pellet was recollected and added to a fresh 100 mL of LB broth and incubated at 37°C. One mL of sample was collected every 5 min for up to an hour. Each collected sample was centrifuged immediately at 10,000 X g for 30 s, and bacteriophage titer was determined from the supernatant. The burst size was estimated by dividing the average number of phages adsorbed on bacteria by the mean of liberated phage particles after bacterial cells burst. The duration required by phages to burst the bacterial cell, resulting in a higher titer, was taken as latent time.

### 2.14 Morphological examination by transmission electron microscope (TEM)

Transmission electron microscopy was performed at the University of Leicester, the United Kingdom, by applying 10 µL of phage stock to the glow discharge 200 mesh formvar/carbon-coated copper grid. After 2 min, the excess fluid was wicked off, and then the grid was washed with 10 µL of water for 30 s and again wicked off with filter paper. Phages were stained by applying 10 µL of 1% (w/v) uranyl acetate for 30 s. The grid was air-dried and then visualized with a JEM-1400 TEM (JEOL UK, United Kingdom) with an accelerating voltage of 100 kV. The images were captured with a Megaview III digital camera and processed in ImageJ to incorporate the indigenous scale bar and average size determination.

### 2.15 Whole-genome sequencing of bacteriophage genome

The phage genome was extracted using PEG-NaCl concentrated phage preparation using a phage DNA isolation kit (Norgen Biotek Corporation, Cat. # 46,850) and quantified by a nano-drop spectrophotometer. Purified DNA was further subjected to DNase I and RNase treatment to confirm the DNA or RNA nature of the phage genome. Digestion with S1 nuclease was carried out to confirm that either phage contains double-stranded or single-stranded DNA. Purified DNA was sequenced with the Illumina NovaSeq 6000 at Macrogen Korea**.** Low-quality reads were trimmed with Trimmomatic. A K-mer analysis was done for the genomic survey, and cleaned, qualified reads were assembled with SPAdes (Bankevich et al., 2012) to get a single consensus contig.

### 2.16 Genome annotation

Open reading frames (ORFs) were predicted with GeneMarkS (Besemer et al., 2001) and the RAST server (Overbeek et al., 2014). To further confirm the predicted ORFs, Shine Dalgarno Sequences were detected with the PECAAN program (https://discover.kbrinsgd.org/autoannotate/). Interproscan (http://www.ebi.ac.uk/interpro/search/sequence-search), NCBI blastp, RAST (Aziz et al., 2008), Pfam (Mistry et al., 2020), HHpred (Zimmermann et al., 2018), HMMER (Potter et al., 2018), CATH and UniProtKB (https://www.uniprot.org/uniprot/) were used to identify the protein domains, families, and to assign functions to predicted ORFs. KEGG was used to predict the genome's molecular functions, cellular components, and biological processes. SignalP (Petersen et al., 2011) and TMHMM (Krogh et al., 2001) were used to predict putative proteins' signal peptides and transmembrane helices. The online tool ARNold was used to detect Rho-independent terminators (Naville et al., 2011). The presence of tRNA was analyzed with the help of Aragorn (Laslett and Canback, 2004) and tRNAScan-SE (Lowe and Chan, 2016). Conserved regulatory elements were identified with the standalone program PHIRE (Lavigne et al., 2004). Phage-specific promotor sequences were identified with PhagePromoter by Galaxy (Sampaio et al., 2019). Codon usage frequency was calculated by the Codon Usage-Sequence Manipulation Suite (https://www.bioinformatics.org/sms2/codon_usage.html). The RepeatMasker web server (https://www.repeatmasker.org/cgi-bin/WEBRepeatMasker) was used to find the interspersed repeats, while the Tandem Repeat Finder (Benson, 1999) was used to locate tandem repeats. The CRISPR finder tool (https://crisprcas.i2bc.paris-saclay.fr/CrisprCasFinder/Index) was used to find the CRISPR-associated genes in JKP2 genome. EffectiveDB (Eichinger et al., 2016) was used to locate putative genes for the prediction of type-III bacterial protein secretion in the phage genome.

### 2.17 Lifestyle and safety prediction

To predict the life cycle of the JKP2, three different online tools were used to find the prophage region in the phage genome. The PHASTER (Arndt et al., 2016) tool was used to identify the intact prophage region, while PHACTS (McNair et al., 2012) and PhageAI (Tynecki et al., 2020) were used to predict the lifestyle of phages. Annotated genome of JKP2 was analyzed manually for the presence of integrase or repressor genes. The genome was uploaded to a resistant gene identifier (RGI) and blast tool incorporated into comprehensive resistance database (CARD) (Alcock et al., 2020) to predict the presence of any antibiotic resistance gene in the phage genome. To detect the integration of any possible host-related virulence factor gene, the JKP2 genome was analyzed by virulenceFinder (2.0) (Joensen et al., 2014), and the virulence factor database (VFDB) (Liu et al., 2019). The presence of Integrative and Conjugative Elements (ICE) was investigated using ICEfinder, a web-based tool (https://bioinfo-mml.sjtu.edu.cn/ICEfinder/ICEfinder.html). The ToxFinder (1.0) software was used to rule out the presence of a mycotoxin synthesis gene in the JKP2 genome. The HostPhinder (1.1) (https://cge.cbs.dtu.dk/services/HostPhinder/) tool was used to confirm the host specificity of JKP2.

### 2.18 Comparative genome analysis and phylogeny

BLASTn was used to find the closest homologs of JKP2. The CJ Biosciences online ANI calculator was used to calculate the average nucleotide identity (ANI) of closest homologs (Yoon et al., 2017). MAUVE progressive multiple sequence alignment (Darling et al., 2004) was used for comparative genome analysis among the closest *Drulisvirus* homologs of JKP2. Comparative analysis of individual ORFs was conducted using CoreGene 3.5 with a 75 threshold (http://binf.gmu.edu:8080/CoreGenes3.5/). For phylogenetic analysis, the putative ORFs for the large terminase and tail fiber protein were compared to other phages with the highest similarity to JKP2 using NCBI Blastp. The retrieved genome data were aligned with MEGA version 10 (Kumar et al., 2018), and a phylogenetic tree was constructed with the UPGMA (unweighted pair group method with arithmetic mean) technique and a bootstrap value of 1000. ViPTree server version 2.0 (Nishimura et al., 2017) was used to compute the proteomic tree for genome-wide differences. ViPTree produces a dendrogram among hundreds of related viruses and can be used to classify new viruses. Genome alignment was carried out to compare the proteomic difference between homologs of JKP2. To classify the virus at the family, genus, and species levels, the Virus Classification and Tree Building Online Resource (VICTOR) tool was used to generate a dendrogram based on whole-genome nucleotide sequence similarities (Meier-Kolthoff and Göker, 2017). It built trees based on the Genome-BLAST Distance Phylogeny method (GBDP).

### 2.19 Analysis for head, neck, tail morphogenesis, and genome packaging strategy

Virfam online tool (Lopes et al., 2014) was used to identify the head, neck, and tail modules of JKP2. Annotated protein sequences were submitted to the Virfam tool to predict structural modules, assign functions to those modules, and determine the morphological types of JKP2. A phylogenetic tree was constructed from the retrieved large terminase subunit sequences of representative phages with known DNA packaging mechanisms to identify the DNA packaging mechanism further.

### 2.20 Statistical analysis

Student unpaired *t*-test and one-way ANOVA incorporated in GraphPad Prism 8.0 were used to compare the JKP2 growth reduction potential against planktonic cells and biofilm of different ages compared to untreated controls. Tukey test was applied for multiple comparisons between groups. A confidence interval of 95% was used to determine statistical significance.

## 3 Results

### 3.1 Characteristics of bacterial strain

A multi-drug resistant *K. pneumoniae* strain 8890 (Kp-8890) was used as a host to isolate the bacteriophage JKP2. It is a multi-drug resistant strain, as determined by the Kirby-Bauer disk diffusion method (Table S1). The strain belongs to the ST-870 clonal group and bears the K-17 capsular serotype and the O1 LPS antigen (Table 1). Several resistance genes were identified in the Kp-8890 genome, including carbapenemase *blaNDM-5, CTX-M-15, blaSHV-106, blaOXA-1, blaTEM-1B*, and *AmpC1*-type beta-lactamases. There were resistant determinants for aminoglycosides *(aac (6′)-Ib-cr, aadA2, aph3-Ia, rmtB, strA, strB)*, fluoroquinolones (*qnrB1, GyrA-83 L, GyrA-87 N*), sulfonamides (*sul1, sul2*), and tetracycline (*tet (A)*). There was no genetic determinant found for colistin, tigecycline, or fosfomycin. Kleborate and VFDB identified key virulence factors like type 1, 3 fimbriae and curli fibres for adhesion, enterobactin siderophore, and yersiniabactin for iron acquisition, capsule for immune evasion, and luxS autoinducer for quorum sensing.

### 3.2 JKP2 produced clear plaques with a large halos

**Fig. 1:**
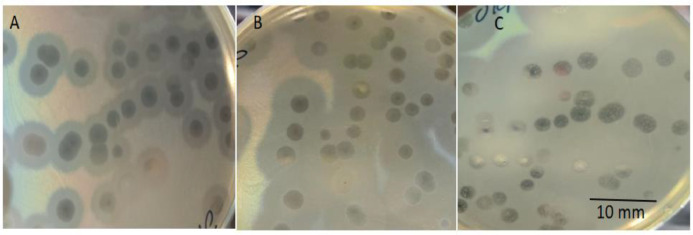
Plaque morphology of JKP2 after (A) 24 h, (B) 48 h and (C) 72 h of incubation at 37 °C on Kp-8890 lawn.

After 24 h of incubation, JKP2 produced bull's eye-shaped clear plaques with a 4–5 mm diameter, surrounded by an opaque halo of 4–5 mm, on the host bacterium. Further incubation expanded the size of the opaque halo to 10–12 mm after 72 h. Resistant colonies appeared in clear plaques after 48–72 h (Fig. 1). JKP2′s optimal MOI was 0.1, and this MOI was used for subsequent characterization studies as and where needed. An average phage titer was calculated after three independent double-layer agar experiments and was found to be 2.35 × 10^9^ pfu/mL.

### 3.3 JKP2 has a narrow host range

JKP2 was found active against four distinct strains of *K. pneumoniae* in a spot test assay (Kp-8890, Kp-9818, Kp-36, and Kp-37). These strains belong to the K-17 capsular serotype. MLST analysis revealed that three of these strains belong to the ST-870 clonal group and one (Kp-36) to the ST-395 clonal group. In contrast, JKP2 exhibited no activity against the other *K. pneumoniae* serotypes or other genera of bacteria tested. Spot test positive phages produced plaques with high virulence, as evidenced by EOP analysis (Table 1).

### 3.4 JKP2 retarded bacterial growth efficiently

**Fig. 2:**
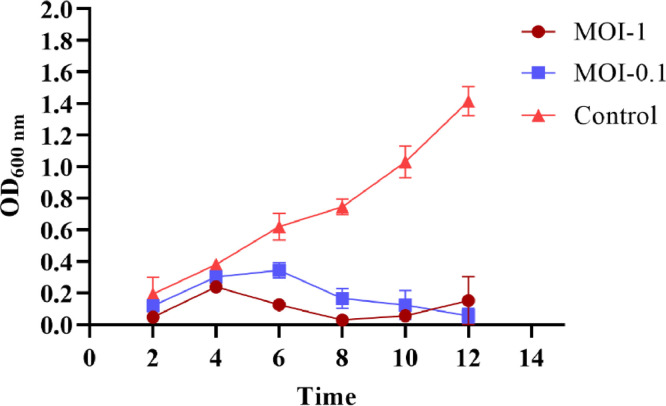
Demonstrating growth reduction potential of JKP2 at MOI-1 and 0.1 compared to control. SEM can be observed by error bars.

**Fig. 3:**
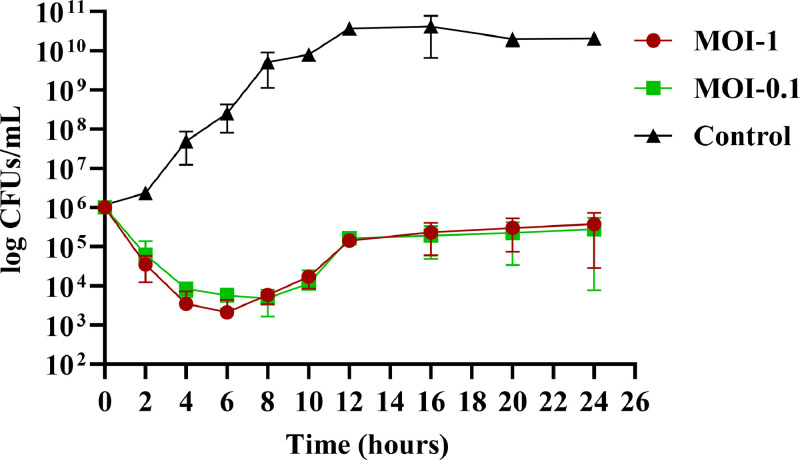
Time kill curve of JKP2 at MOI-1 and 0.1 compared to zero-time point and untreated growth control. SEM can be observed by error bars.

JKP2 controlled bacterial growth for 12 h at both MOIs (0.1, 1) compared to the control. MOI-1 was found more effective than MOI-0.1 in terms of bacterial reduction (Fig. 2). The difference in OD_600_ between the control and JKP2 treatments was statistically significant (p-value = 0.0001). A time-kill study showed that JKP2 reduced bacterial growth at MOI-1 and 0.1 with equal efficacy (t-test p value >0.05). After two hours of treatment, the log reduction was 1.45 and 1.20 at MOI-1 and 0.1, respectively. At both MOIs, bacterial population was reduced by more than 2-logs after 4, 6, and 8 h of treatment. After 8-hours of treatment, bacterial counts started to rise, although the decrease was still greater than 2-log, which dropped to 1.7-log at MOI-1 and 1.9-log at MOI-0.1 post 10 hour treatment. After 12 h of phage challenge treatment 0.84 and 0.78-log reduction was observed at MOI-1 and 0.2, respectively. After completion of 24 h of phage challenge, 0.42 and 0.55-log reduction in bacterial count was observed (Fig. 3). Frequency of Kp-8890 BIMS was found to be 1.02 ± 0.34 × 10^−6^ at MOI-1 while 1.73 ± 0.51 × 10^−6^ at MOI-0.1.

### 3.5 Kp-8890 established mature biofilm at 96 hours’ post-inoculation

**Fig. 4:**
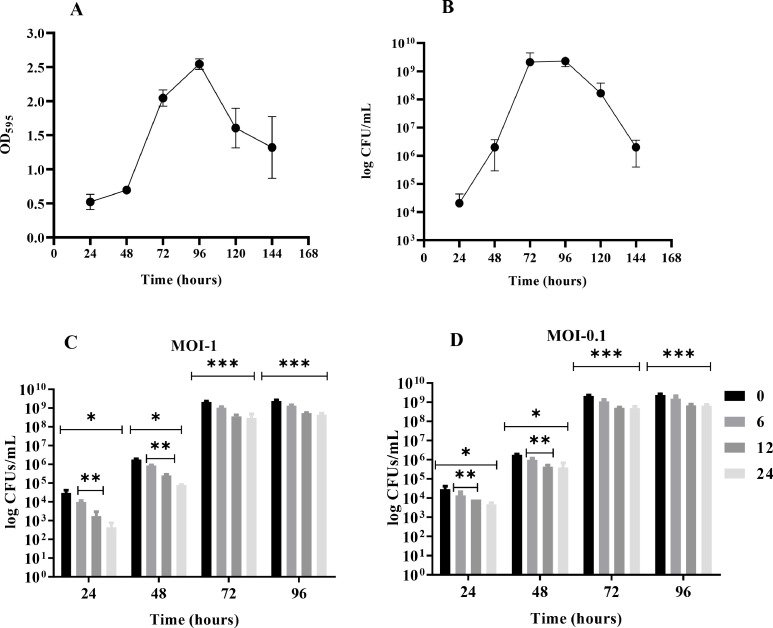
Figure 4A and B are showing *K. pneumoniae* biofilm formation kinetics study by CV assay (A) and viable count assay (B). While figure C and D demonstrating the biofilm removal in log CFUs/mL after JKP2 treatment for 6, 12 and 24-hours for 1–4 days old biofilms at MOI-1 (C) and 0.1 (D) in comparison to zero-time point. The bars marked with * show a statistically significant reduction in biofilm at each treatment time point (6, 12, and 24-hour) compared to the zero-time point. The bars marked with ** indicate a statistically significant difference in biofilm reduction between the 6 and 12-hour treatment periods. While, the bars marked with *** demonstrate a statistically significant difference in biofilm reduction only after 12 and 24-hour treatment compared to the zero-time point. For determining statistical significance a threshold of a p-value <0.05 was employed.

The maturation stages of biofilm were determined by CV staining methods and the viable cell count method. At 24 h of incubation, Kp-8890 established biofilm, resulting in 2.0 × 10^4^ CFUs/mL. Maximum CFUs/mL was produced at 96-hours post-inoculation, which resulted in 2.3 × 10^9^ CFUs/mL. Similar results were observed for the CV method, where the highest biomass was stained at 96 h with an OD_595_ value of 2.54. One and three log reductions in the bacterial count were found on the 5th and 6th day of biofilm growth, respectively (Fig. 4).

### 3.6 JKP2 significantly removed mature biofilm

The JKP2 biofilm removal capability was assessed by comparing the log reductions in cell counts after phage treatment with the cell counts at zero-time points. While the biofilm removal and controlling ability were also assessed by comparing the cell counts with untreated biofilm controls. An average log reduction was calculated and will be discussed hereafter. Individual p values for every statistical analysis are shown in table S2 and S3.

**Fig. 5:**
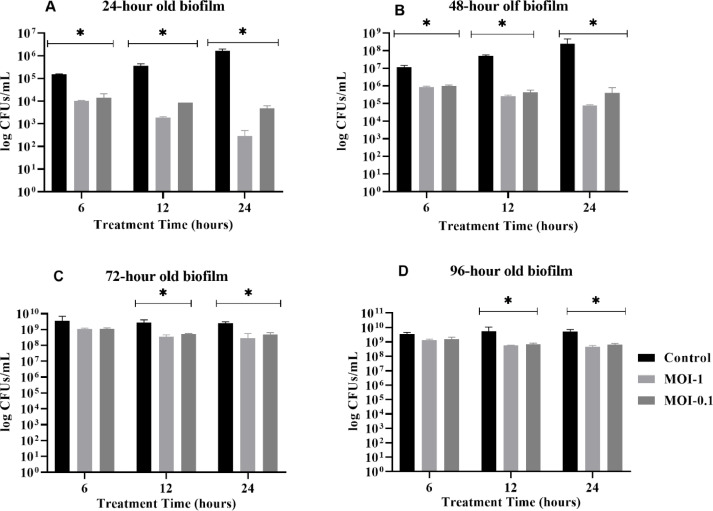
Biofilm removal in log CFUs/mL after JKP2 treatment for 6, 12 and 24-hours for 1–4 days old biofilms at MOI-1 and 0.1 in comparison to untreated biofilm growth control. Bar with * indicate statistically significant post treatment reduction in relation to untreated biofilm growth control.

After 6, 12, and 24 h of phage treatment at MOI-1, JKP2 eliminated 0.429 (64%), 1.193 (94%), and 1.783 (98%) log biofilm population from a 24-hour-old biofilm (Fig. 4C). For 24-hours old biofilm, phage treatment at MOI-1 in comparison to the untreated control, resulted in log reductions of 1.177 (94%), 2.327 (100%), and 3.567 log (100%) at 6, 12 and 24 h post-treatment, respectively (Fig. 5A). There was a statistically significant difference in bacterial counts observed after 6, 12 and 24 h of phage treatment when compared to zero-time point or untreated bacterial counts (p value <0.05).

At MOI-1, JKP2 removed 0.365 (53%), 0.884 (86%), and 1.402 (96%) log bacterial population from 48-hour-old biofilm after 6, 12, and 24-hours of phage treatment, respectively (Fig. 4C). For 48-hours old biofilm, phage treatment at MOI-1 in comparison to the untreated control, resulted in log reductions by 1.116 (94%), 2.283 (99%), and 3.500 (100%) at 6, 12 and 24 h post-treatment, respectively (Fig. 5B). Statistically significant biofilm reduction was observed at all-time points in comparison to zero-time point or untreated bacterial counts (p value <0.05).

JKP2 biofilm removal efficiency was reduced for 72- and 96-hour-old biofilms. JKP2 removed 0.307 (49%), 0.767 (82%), and 0.854 (86%), respectively, of the log bacterial population from 72-hour-old biofilm after 6, 12, and 24-hours of phage treatment at MOI-1 respectively (Fig. 4C). Somewhat similar results were observed against 96-hours old biofilm as JKP2 removed 0.244 (48%), 0.625 (78%), and 0.718 (82%) log bacterial population after 6, 12 and 24-hours of phage treatment at MOI-1 respectively (Fig. 4C). A 6-hour phage treatment did not result in a statistically significant reduction in the biofilm population for 72- and 96-hour-old biofilms. However, 12 and 24-hour phage treatment resulted in a statistically significant reduction in 72 and 96-hour old biofilm (Fig. 4C and D). Somewhat similar results were obtained when compared with untreated biofilm growth control (Fig. 5). Figure S1 is showing CV-stained biofilm at different time points.

At any time-point, there was no statistically significant difference in biofilm removal was observed between MOI-1 and 0.1 (individual p values for specific time-points are given in table S2 and S3). Moreover, phage treatment for 12 or 24 h did not differ significantly regardless of the age of the biofilm (p value >0.05).

### 3.7 JKP2 was stable at various physiochemical conditions

**Fig. 6:**
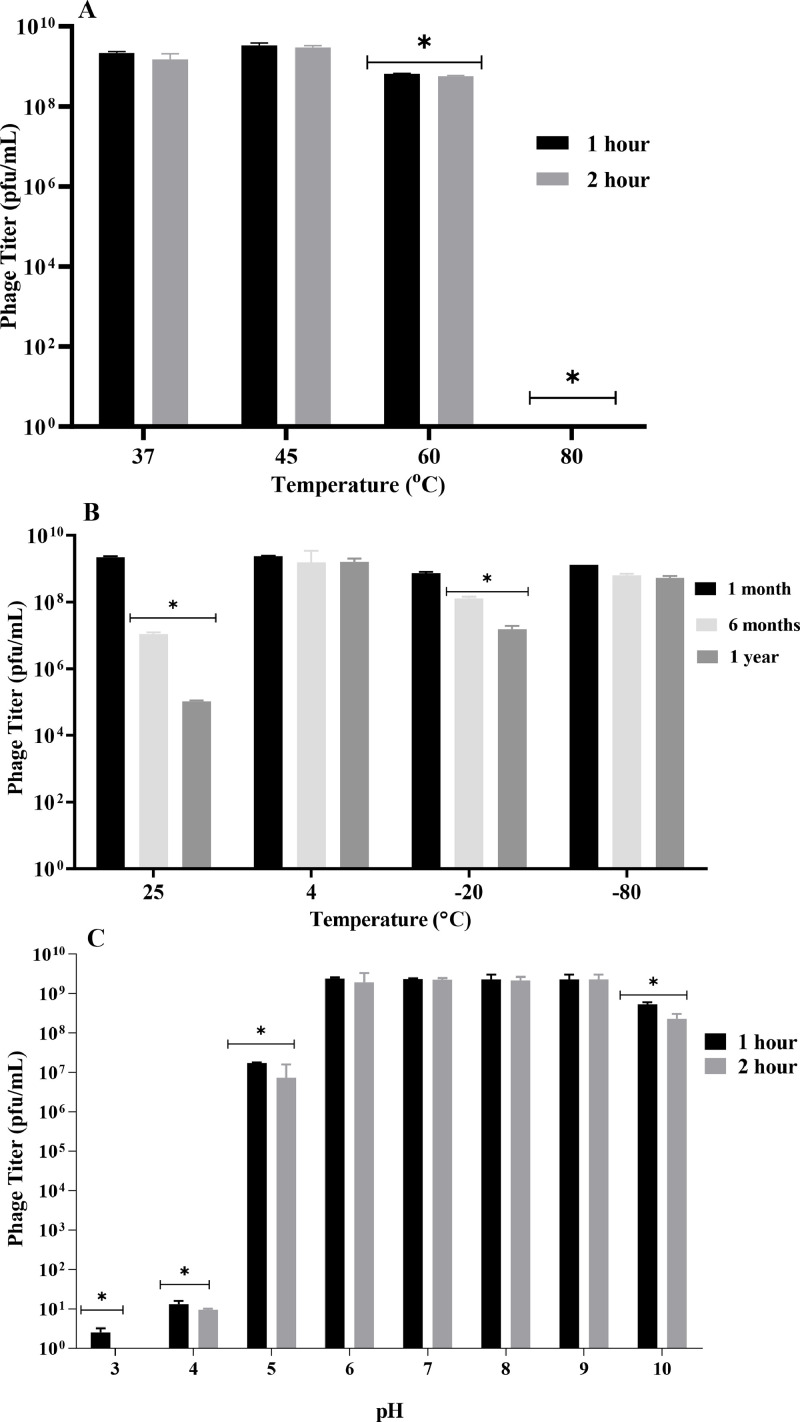
JKP2 stability after 1 and 2 h of heat treatment (A), long-term storage stability at various temperatures at different time intervals, and (B), the effect of various pH treatments on JKP2 stability (C). The mean titer after three independent experiments is shown as a bar graph. Error bars indicate SEM. Bar with * indicate significant reduction in titer relative to zero-time point (p value <0.05).

JKP2 treatment at pH 6–9 for 1–2 hour showed insignificant change in titer (p value >0.05). Treatment at pH 5 and 10 resulted in 2 and 1 log reductions in titer, respectively, which was also statistically significant (p-value <0.05). While at pH 3 and 4, few plaques were produced (p value <0.05). Heat treatment at 37 and 45 °C produced statistically insignificant effect on phage titer; however, at 60 °C, 1-log reduction (p vaue <0.05) in phage titer was observed while at 80 °C, JKP2 was completely inactivated. The JKP2 titer was insignificantly affected by one-month storage at all temperatures tested (4, 25, −20, and −80°C) (p value >0.05). After six months of storage, JKP2 remained stable at 4 and −80 °C while titer decreased significantly by 1 and 2 logs at −20 and 25°C, respectively (p value <0.05). Storage at 4 and −80°C did not influence the titer significantly after one year (p value >0.05) (Fig. 6).

### 3.8 JKP2 has an extended latent period and short burst size

**Fig. 7:**
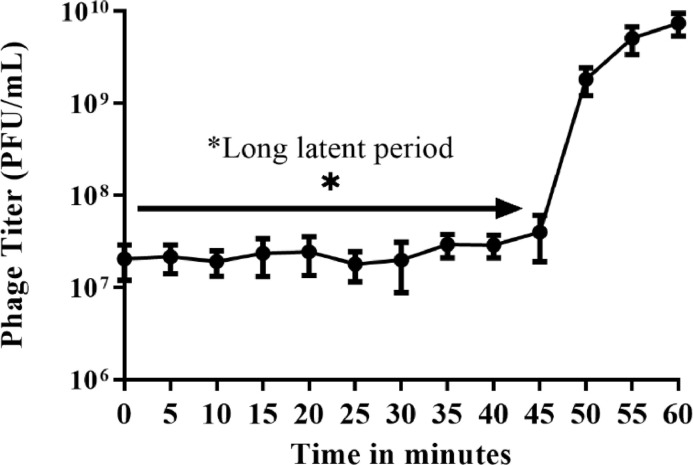
One-step growth curve kinetics of *Klebsiella* phage JKP2. Abrupt increase in phage titer is evident after 45 min.

JKP2 has a latent period of 45 min. The burst occurred between 45 and 50 min and produced 70 PFU/cells (Fig. 7).

### 3.9 JKP2 has a morphological resemblance with podoviruses

**Fig. 8:**
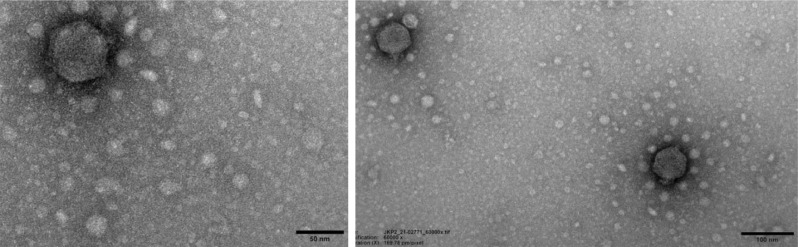
Transmission electron micrographs of JKP2, showing icosahedral capsid with a short non-contractile tail.

The transmission electron micrograph of JKP2 demonstrated an icosahedral capsid with an estimated diameter of 54 ± 0.5 nm and a short, non-contractile tail, measuring 12 ± 0.2 nm, designating it as a member of the old *Podoviridae* family (Fig. 8).

### 3.10 Genome annotation

**Fig. 9:**
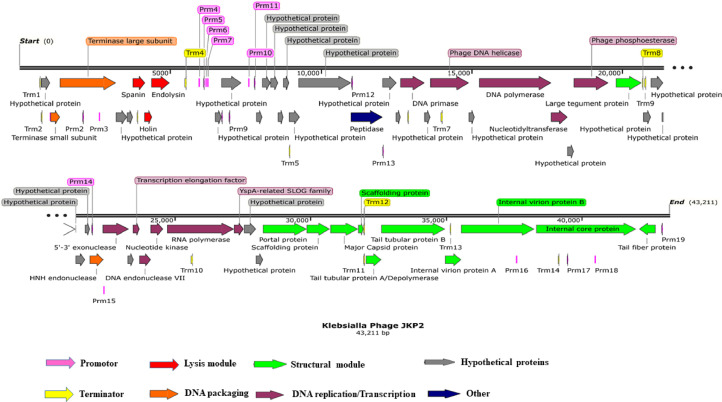
Linear genome map of JKP2 drawn with SnapGene 6.0. Putative ORFs and regulatory sequences are shown with different color arrows, representing functional modules of the genome. The direction of the arrows represents the transcription direction. Among 54 ORFs, 30 ORFs with known functions and 24 ORFs with hypothetical functions are shown here, 19 promotors and 14 rho independent terminators are also shown. Abbreviations: Prm; Promotor, Trm: Rho independent terminator

No digestion of extracted DNA genome with, RNase, and S1 nuclease and complete digestion with DNase I confirmed that JKP2 has double-stranded DNA as its genome. NGS results showed that it has a 43,211 bp genome with 54.1% GC content. GeneMarkS predicted 55 ORFs, while the RAST server predicted 59 coding DNA sequences. The ORF was not considered a gene if the CDS of the putative ORF was shorter than 120 bp or if the PEECAN software has not detected the Shine-Dalgarno Sequence (SDS). ORF 1 predicted by RAST and GeneMarksS was excluded as SDS cannot be detected for this ORF, therefore, 54 ORFs were considered for further analysis. All ORFs were present on the plus strand except ORF 54. Annotation with different databases showed that 29 ORFs (54%) encode proteins with already known functions, while 25 ORFs (46%) were termed as hypothetical proteins as no homologs could be found in genomic databases.  ORF 53 was the largest gene that comprises 3396 bp and encodes for an internal core protein, while ORF 20 (147 bp) encodes for the smallest protein with a hypothetical function. Genes for DNA packaging enzymes were clustered at the start of the genome, followed by the genes encoding three-step lysis cassettes viz holin, endolysin, and spanin. Genes for replication and transcription-related enzymes/proteins were scattered throughout the genome, but relatively, they were clustered in the middle of the genome. Genes for structural proteins were clustered at the end of the genome (Fig. 9 and Table S6 and S6.1).

Signal peptide sequences were detected in four putative genes. One of them encodes a hypothetical protein (ORF22), while the others (ORF6, 8, and 51) encode for spanin, endolysin, and internal virion protein, respectively (Table S6.1). Two transmembrane helices (TMH) were detected in putative spanin and endolysin encoding genes (ORF6 and 8), while one TMH was detected in holin (ORF7). Three ORFs encoding hypothetical proteins also showed one TMH (Table S6.1). PhagePromotor identified 19 promotor sequences; only four were phage-specific, while others were host-specific (Table S7). A total of 14 Rho-independent terminators with various loop lengths and stems were also detected in the JKP2 genome (Table S8). The PHIRE algorithm discovered conserved regulatory elements for large tegument protein (ORF29), portal protein (ORF45), internal core protein (ORF53), and nucleotidyltransferase (ORF26). The consensus sequence for the regulatory element was AGCAGCTCCAGCAGCTCCAG. No specific function, like promotor, terminator, or *Ori*, was ascribed to the regulatory sequence.

Codons CTG and GCA were the most commonly used codons (26.87 and 26.73/1000 codons), and they encode leucine and alanine, respectively. Codon TTT was the least used codon (7.29/0.33), and it codes for phenylalanine. No tRNA or rRNA was detected in the JKP2 genome.

No long terminal repeat (LTR), long interspersed nuclear elements (LINE), short interspersed nuclear elements (SINE), or DNA elements were detected in the JKP2 genome by the Repeat Masker tool, but two simple satellite repeats were detected (Figure S2). The Tandem repeat finder also detected two repeats spanning 12 and 9 nucleotide repeats with repetitions of 2.3 and 3.6 (Figure S3). No CRISPR-associated genes were detected in the JKP2 genome. Effective T3 software predicted six ORFs with type III secretion proteins (Figure S4). The annotated genome was submitted to NCBI under accession number ON165415.1.

Phage JKP2 encodes a range of enzymes involved in DNA metabolism. It encodes primase (ORF21) and helicase (ORF23) to initiate DNA replication. For replication, it uses DNA-directed DNA polymerase (ORF25) and 5′−3′ exonuclease (ORF36) to remove ribonucleotide primers and DNA repair, while for transcription, it also encodes DNA-directed-RNA polymerase (ORF41). HHpred identified a SPT5-like transcription factor (ORF38) that acts as a clamp between DNA and RNA hybrids to initiate transcription. ORF26 encodes CCA adding nucleotidyl transferase that helps in post-transcriptional modifications of pre-mRNA by catalyzing the transfer of cytidine and adenosine monophosphate at the 3′ end. ORF42 encodes a protein belonging to the Yspa-related-SLOG family that acts as a sensor for nucleotide buildup and is also involved in NAD metabolism.

The JKP2 phage encodes a three-step cassette lysis system. ORF 6, 7, and 8 encode spanin, holin, and endolysin, respectively. These ORFs exhibit varying degrees of genomic overlap. The spanin and endolysin gene products both contain a signal peptide sequence and two transmembrane helices. Endolysin of JKP2 comprises a single globular domain and it belongs to the lysozyme domain superfamily. The holin protein of JKP2 (ORF7) is comprised of 83 amino acids and shows a non-cytoplasmic domain at the N-terminus and a cytoplasmic domain at the C-terminus, while one transmembrane helix was identified in the center of the protein sequence. It has a charged and hydrophobic C-terminus.

### 3.11 JKP2 is safe for therapeutic application

No intact prophage was detected in the JKP2 genome by PHASTER. PhageAI and PHACTS confidently predicted its lytic nature. Antibiotic resistance genes, bacterial virulence factors, mycotoxins, and integrative and conjugative elements were not detected in the JKP2 genome. Phage encoded integrase, excisoinase, and repressor genes could not be located even after complete annotation with various databases. *K. pneumoniae* was designated as its host based on genomic analysis by the HostPhinder tool.

### 3.12 JKP2 belongs to the *unclassified Drulisvirus* of the *Autographiviridae* family

According to BLASTn and ANI calculations, the closest homologs of JKP2 are Klebsiella phage NER40, phiKpS2, Bp5, SU503, and KpV41, which share 90–93 percent homology with 83–89 percent query coverage. All of these phages are classified as *unclassified Drulisvirus* within the *Autographiviridae* family. JKP2 has a genome length of 43 Kb, comparable to other family members having a 42–44 Kb genome with 50–58 genes and GC content of around 54%. No GC skew was observed in the JKP2 genome, indicating unidirectional replication. (Figure S5).

**Fig. 10:**
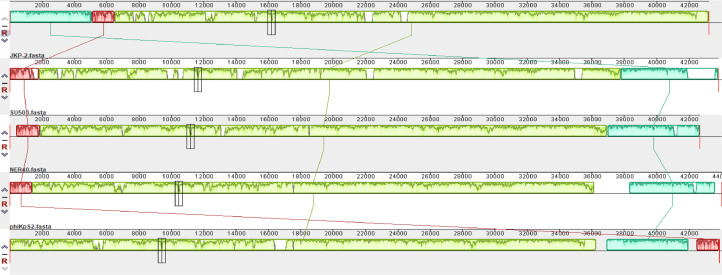
Mauve multiple sequence alignment with JKP2 closet homologs. Block colors indicate areas of homology with no internal rearrangement. Block location above the central line indicates the forward orientation of all genes. White color gaps indicate novel regions.

**Fig. 11:**
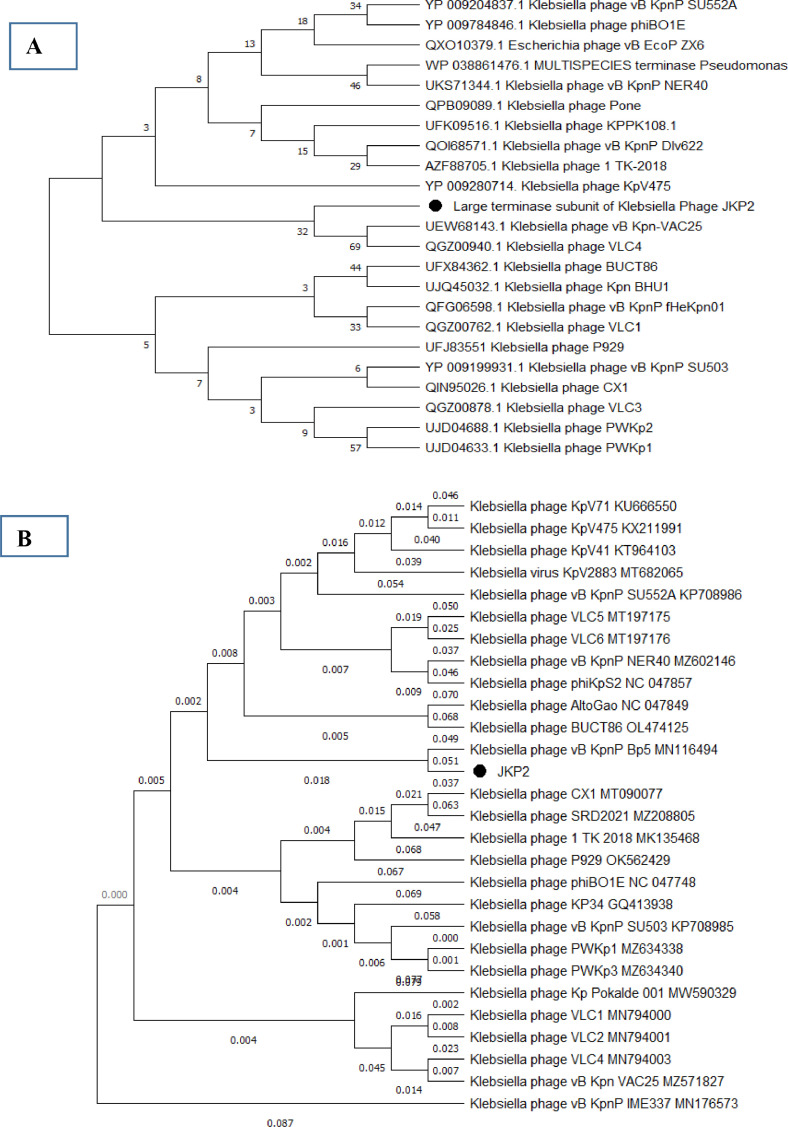
Phylogenetic tree of large terminase subunit (A) and whole-genome sequences (B) of JKP2 by MEGAX and VICTOR. UPGMA method with 1000 bootstrap value was employed for tree construction in MEGAX, while the default setting of VICTOR was used to construct a whole-genome tree.

**Fig. 12:**
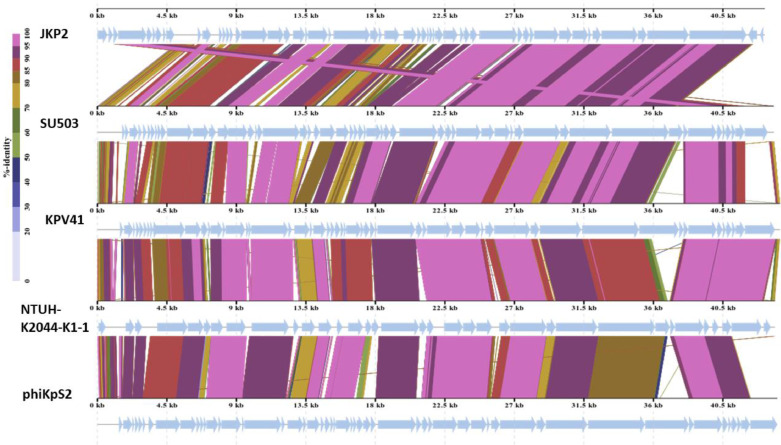
Comparative analysis of JKP2 with four closely related Klebsiella phages. Genome alignment was created by the VipTree tool. Highly conserved regions are shaded as pale pink. Arrows indicate the direction of transcription for the predicted ORFs. The termini of the JKP2 genome were entirely different relative to its closely related phages.

Multiple sequence alignment with MAUVE indicated that JKP2 and its closet homologs showed three homolog blocks, but their location varied among genomes, indicating genetic rearrangement. All of the aligned regions showed forward orientation (Fig. 10). Phylogenetic analysis of the JKP2 large terminase subunit revealed its close relationship to Klebsiella phages VAC25 and VLC4, whereas a phylogenetic tree was constructed from the complete genome sequence revealed its relationship to Klebsiella phage Bp5 (Fig. 11). These closely related phages are members of the family *Autographiviridae*, the subfamily *Slopekvirinae*, and the unclassified genus *Drulisvirus*. The JKP2 was also classified as a member of the *Autographiviridae* family according to a proteomics-based dendrogram constructed by the VipTree program (Figure S6). VipTree obtained genome-wide alignment of JKP2 with closely related phages showed high similarity among related genomes. However, the terminal portion of the genome was grossly different compared to related genomes (Fig. 12).

### 3.13 JKP2 uses a T7-like direct terminal repeats mechanism for DNA packaging

**Fig. 13:**
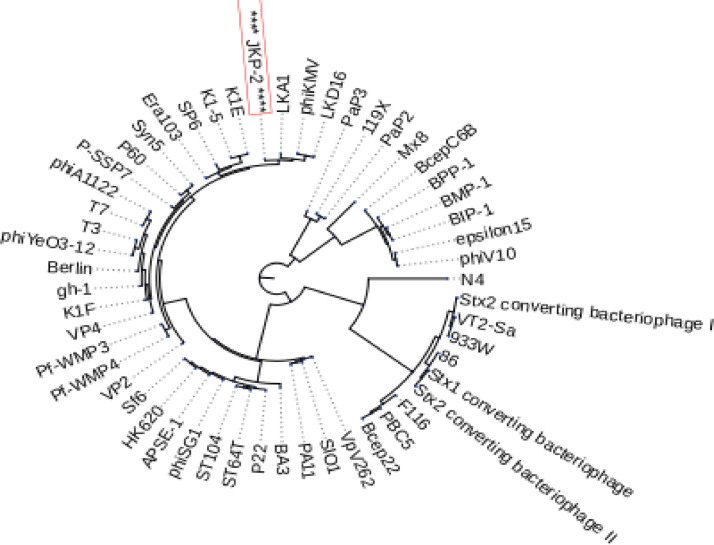
Virfam generated proteomic tree of JKP2 based on the head-neck-tail module. JKP2 made a cluster with LKA1 and PhiKMV, T7-like *Pseudomonas* phages.

**Fig. 14:**
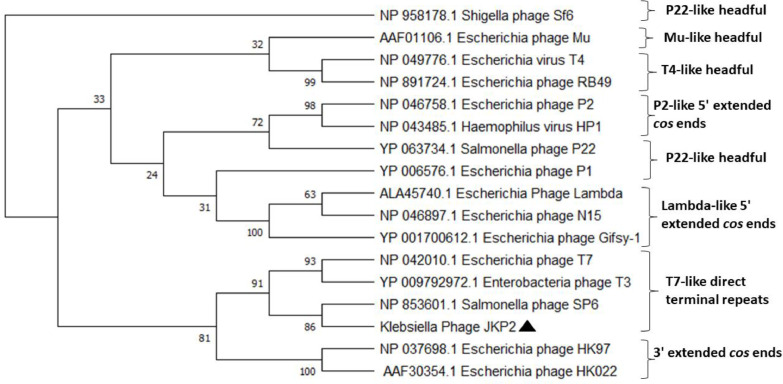
Phylogenetic tree of a terminase large subunit of representative phages with known DNA packaging mechanism and JKP2. The tree was constructed by MEGAX, using the UPGMA method with a 100 bootstrap value. .

The Virfam tool classified the JKP2 as a type 3 *Podoviridae* that display structural similarities with Salmonella phage P22. Two separate scaffolding proteins (ORF46 and 48) and a major capsid protein (MCP) (ORF47) were recognized in the JKP2 genome. Virfam tool identified the large terminase subunit (ORF3) while the small terminase subunit was not identified by it. It designated ORF 45 as a portal protein that acts as a ring-shaped dodecamer to transfer the DNA into the procapsid. Type 3 adapter protein (ORF 49) and head closure protein (ORF50) were also detected (Figure S7). The Virfam clustered the JKP2 with PhiKMV and LKA1, T7-like phages based on a head-neck-tail organization (Fig. 13). A phylogenetic tree was constructed from the retrieved large terminase subunit sequences of representative phages with known DNA packaging mechanisms to further speculate on the DNA packaging mechanism. JKP2 clustered with T3, T7, and Sp6 phages that use the T7-like direct terminal repeats mechanism for DNA packaging (Fig. 14).

## 4 Discussion

In this study, a bacteriophage JKP2 was isolated and characterized against the K-17 serotype of *K. pneumoniae*, a locally prevalent, hyper-virulent ST-870 strain. This is the first report describing the characterization of phage against the K-17 serotype to the best of our knowledge. The phage JKP2 exhibited large clear plaques with a semi-transparent halo around them. This could be due to the presence of phage-encoded capsular depolymerase, which lyses the extra-polymeric substance of the bacterial capsule to reach the outer membrane receptor. These capsular depolymerases are usually part of the tail proteins or sometimes secreted separately (Pyra et al., 2020). JKP2 showed a very narrow host range and infected only the K-17 serotype like its closest homologs, Klebsiella phage NER40 and Bp5, which infected K-2 and K-20 serotypes, respectively (Gorodnichev et al., 2021; Zhang et al., 2021a). Klebsiella phages often show a narrow host range with serotype specificity. Bacteriophage NTUH-K2044-K1–1 infects capsular type K-1 and SH-KP152226 infects K-47 strains only (Lin et al., 2014; Wu et al., 2019). The JKP2 lysed K-17 serotype with high virulence as indicated by the EOP test regardless of within serotype diversity. Although interspecies narrow host range warrants an advantage of targeted therapy, its intraspecies narrow host range is also a hurdle in the application of Klebsiella phages.

JKP2 controlled *K. pneumoniae* planktonic cells till 12 h of incubation at MOI-1 and 0.1 with equal efficacy. Time-kill study showed that JKP2 has moderate bacterial killing efficiency. The reduction was maximum during 2–10-hours post phage challenge. The resistant mutants started to accumulate after 8-hours of phage challenge and after 24-hours, only 0.42 and 0.55-log bacterial reduction was observed with respect to zero-time point. It reduced bacterial growth at MOI-1 and 0.1 with equal efficacy (t-test p value >0.05). Exposure of phages to their respective host results in emergence of BIMS most commonly due to acquired point mutations in genes encoding receptor molecules. High frequency of BIMS may undermine the efficacy of phages against their respective host (García et al., 2007). Kp-8890 showed moderate frequency of BIMs (1.02 ± 0.34 × 10^−6^ at MOI-1 while 1.73 ± 0.51 × 10^−6^ at MOI-0.1) against JKP2 phage. In this study higher BIMS frequencies were observed at MOI-0.1 in comparison to MOI-1 and the difference in BIMs frequency at MOI-1 and 0.1 was statistically significant (t-test p value <0.0001). When phages are used in low doses, a lower immune response is generated (Asif et al., 2020), whereas higher MOI challenges results in the emergence of bacteriophage-insensitive mutants (Mosterd et al., 2021). However, Many factors affect the frequency of BIMS development like bacterial strains, nature of phage (lytic or lysogenic), evolutionary pressure, bacterial heterogeneities and MOI (García et al., 2007). Low MOI imparts more time to bacteria for possible co-evolution. Furthermore, at low MOI, the phages may not be able to completely eliminate the susceptible bacteria in the population, allowing for their survival and transformation to resistant mutants (Bull et al., 2014). Phage-host interaction studies may shed more light on co-evolution of bacteria and phages at different MOIs.

Therefore, for therapeutic purposes, a lower MOI with minimum efficacy is more appropriate. *K. pneumonia.* Kp-8890 genome analysis indicated that it has genetic determinants for biofilm formation such as type 1 and 3 fimbriae that provide adhesion to surfaces. It also possesses genes to produce extra-polymeric substances where biofilm-producing bacteria are embedded. Kp-8890 produced biofilm of 1–4 days was treated with JKP2. After 6-hours of phage administration, JKP2 effectively removed 64% and 49% of Kp-8890-produced 24-hours old biofilms at MOI-1 and 0.1, respectively. However, its activity was time-dependent as 12 h of treatment resulted in 94% and 70% biofilm removal at MOI-1 and 0.1 respectively. Similar pattern was observed for 48-hours old biofilm. However, 72 and 96-hours old biofilm was not removed significantly after 6-hours of phage treatment instead required 12 or 24-hours of phage treatment for significant removal. Treatment time for 6-hours and 12-hours produce statistically significant difference in biofilm removal against 24 and 48-jpurs old biofilm, however, no significant difference was observed between 12 and 24-hours of treatment. Although the biofilm removal was lower with MOI-0.1 in comparison to MOI-1 at all time-points, the difference was not statistically significant. As the biofilm matured, JKP2′s efficiency dropped but it still managed to eliminate 82% and 74% of 96-hours old biofilm at MOI-1 and 0.1 respectively, after 24-hours of phage treatment.

Although biofilm reduction by JKP2 was statistically significant, still high burden of biofilm was left behind ranging from 10^4^-logs for 24 h biofilm and 10^7^-logs for 72–96-hours biofilm in comparison to untreated control. It shows that JKP2 has moderate activity against young biofilm and modest activity against mature biofilm when used alone. It is already been reported that most phages alone had a modest effect in eliminating biofilm (Townsend et al., 2020). However, when applied simultaneously with antibiotics or other synergistic agents like DNase enzymes (Ku et al., 2021), iron antagonizing compounds (Chhibber et al., 2013), and phage-derived lytic proteins (Duarte et al., 2021), a significant improvement in the killing effect was observed. Although synergistic activity of JKP2 with antibiotics or other agents were not examined in this work, the possibility of increased efficacy can be anticipated. Reduction in biofilm has also been attributed to the soluble production of capsular depolymerase. It actively degrades the EPS, enabling phages to attach to bacterial cells (Ku et al., 2021). The modest biofilm removal by JKP2 may be due to the expression of depolymerase or slowly replicating phages against mature biofilm, but it cannot be stated unless confirmed otherwise. However, in this study, the impact of depolymerase on biofilm removal was not evaluated separately.

Newly isolated phages should be evaluated for their stability and persistence when exposed to a variety of external conditions. JKP2 remained stable at pH values ranging from 5 to 10 and temperatures ranging from 37 to 60 °C. However, its titer was significantly reduced following one-hour treatment at pH 4 and a temperature of 60°C (Fig. 6). This could be due to the denaturation of phage proteins at high temperatures. Phages withstand better in neutral to alkaline conditions and aggregate in extremely acidic conditions resulting in loss of activity, as seen in Escherichia phage MS2 (Asif et al., 2021; Tabassum et al., 2018a). This study established that JKP2 can be stored in SM buffer at room temperature for one month, but 4°C and −80°C are optimal temperatures for longer storage. Storage at −20°C showed 2-log reduction in JKP2 phage (Fig. 6). This may be due to slow freezing at −20°C resulting in large ice crystals formation that affects the phages viability (Gonzalez-Menendez et al., 2018). JKP2 has a latent period of 45 min with an intermediate burst size of 70 pfu/cell. JKP2 homolog phage phiKpS2 has a 90-minute latent period, whereas Bp5 has 40 min with 269 and 40 phages per cell, respectively (Solovieva et al., 2018; Zhang et al., 2021b). It has previously been demonstrated that phages with intermediate burst times exhibit the highest fitness and hence may be more useful for therapeutic purposes (Asif et al., 2021).

According to the new ICTV classification method, Klebsiella virus JKP2 is categorized as a member of the *unclassified Drulisvirus* genus under the *Autographiviridae* family. It shares more than 90% homology with Klebsiella phage NER40, phiKpS2, Bp5, SU503, and KpV41 of *Drulisvirus* within the *Autographiviridae* family. Its GC content (54.1%) and genome length are comparable with other members of the genus. Although lytic phages typically have a lower GC content than their host bacteria, JKP2 host strain Kp-8890 had a nearly identical GC level of 54.7% (Khan et al., 2021).

No tRNA or rRNA was detected in the JKP2 genome. Due to codon biases, most lytic phages typically encode their tRNA, especially for rare codons (Alvi et al., 2021). JKP2 is a lytic phage as shown by lytic plaque morphology and the absence of integrase or repressor genes. The whole-genome sequence study of *Klebsiella* strain Kp-8890 revealed that the GCG codon (35.87/1000 codons) was the most frequently used codon that encodes for alanine. JKP2 uses host tRNA and has codon bias for GC-rich codons just like its host. This may be reason that GC content of JKP2 phage and its host strain is almost equal despite being a lytic phage.

Phage JKP2 encodes a range of enzymes involved in DNA metabolism. Sequences for these encoding genes are conserved among members of the genus *Drulisvirus*. The JKP2 phage encodes a three-step cassette lysis system that lacks the canonical order of lysis genes and contains a lysis cassette characteristic of T7-like phages (Xu et al., 2021). Its close homologs, NER40 and phiKpS2, also have the same structural organization (Gorodnichev et al., 2021; Shen et al., 2018). HHpred showed the high similarity of ORF8 with R21 endolysin of coliphage P21 with probability of 99.71% and e-value of 2e-15. This R21 has N-terminal signal-anchor-release (SAR) domain (Sun et al., 2009). Furthermore, the presence of a signal peptide in JKP2 endolysin is an indication that it is a signal-anchor releasing endolysin as seen in ϕKMV phage (Briers et al., 2011). In comparison to endolysins, holins are significantly more diversified and frequently unique in terms of amino acid sequence (Catalao et al., 2013). However, JKP2 holin revealed the highest degree of similarity with the Klebsiella phage vB KpnP IME337, which contains a P21-like pinholin (Kongari et al., 2018; Young, 2013). It can be concluded that JKP2 uses pinholins-SAR endolysin and spanin to lyse the cell.

Structural module analysis of JKP2 with Virfam tool revealed more than 97% homology with members of the genus *Drulisvirus*. These phage proteins have conservative folds among different phages despite limited sequence similarity (White and Orlova, 2019). *Podoviridae* phages possess adsorption machinery in the form of short tails for phage adsorption. JKP2 has a short tail and ORF54 encodes a tail fiber protein that is possibly employed to adsorb on the bacterial cell surface. ORF51, 52, and 53 encode internal virion protein A, B, and inner core protein respectively. As described by Chang et al. (2010), in bacteriophage T7, these proteins form a cylindrical structure that is employed to eject the phage genome into the infected cell. ORF49 of JKP2 encodes tail tubular protein A and showed maximum homology with Klebsiella phage KpV475 (98.84%), Bp5 (98.27%) and KP34 **(**97.69%)**.** It serves a dual function, as a structural gatekeeper; and as exopolysaccharidase, as reported in the case of KP34 (Zhang et al., 2021b).

According to the phylogenetic analysis of the large terminase subunit, JKP2 is clustered with phages that use a T7-like direct terminal repeat strategy for genome packaging. Virfam also clustered it with T7-like phages. A study by Casjens and Gilcrease (2009) reported that the large terminase-created genome ends are usually conserved in tailed phages. With a few exceptions, the DNA packaging mechanisms of new phages can be predicted with high confidence by phylogenetic analysis of large terminase with representative phages of known packaging mechanisms (Amarillas et al., 2021; Casjens and Gilcrease, 2009). As a result, it can be inferred that JKP2 used a T7-like direct terminal repeat method to package its genomes.

## 5 Conclusion

JKP2 is a new phage against the K-17 serotype of *Klebsiella pneumoniae*. It has an excellent growth reduction ability against planktonic cells and mature biofilm. Although it is a narrow host range, it has shown high infectivity against specific serotypes. JKP2 has shown stability and activity under varying physiological conditions. It does not possess any integrase or repressor genes, bacterial virulence/toxin, or resistant genes so can be applied safely for therapeutic purposes after *in-vivo* studies.

## Author contributions

This manuscript is a part of Muhammad Asif, PhD thesis and he conducted the majority of the experimental work and drafted manuscript. I.A.A helped in genome analysis. M.W helped in host strains characterization and host range assessment. A.B and F.A.R helped in phage isolation characterization. Dr. S.R. conceptualized and supervised all experiments and manuscript preparation. All authors have reviewed the final version of the manuscript.

## Data availability

The annotated genome was submitted to NCBI under accession number ON165415.1.

## Declaration of Competing Interest

The authors declare no conflict of interest. This research work was conducted as a part of the Ph.D degree of Muhammad Asif.

## Data Availability

Data will be made available on request.

## Appendix Supplementary materials

Image, application 1

Image, application 2
