# Supplementary material for: A K-17 serotype specific Klebsiella phage JKP2 with biofilm reduction potential
Source: Virus Res. 2023 Apr 2;329:199107. doi: 10.1016/j.virusres.2023.199107 (PMC10194101; doi:10.1016/j.virusres.2023.199107)

**A novel K-17 serotype specific *Klebsiella* phage JKP2 with biofilm reduction potential**

**Supplementary DATA**

Muhammad Asif^1,2^, Iqbal Ahmed Alvi^1,3^, Muhammad Waqas^2^, Abdul Basit^1^, Faiz Ahmed Raza^4^, Shafiq-ur-Rehman^1^

**Authors information**

1. Institute of Microbiology and Molecular Genetics, University of the Punjab, Lahore, Pakistan
2. Department of Pathology, King Edward Medical University, Lahore, Pakistan
3. Department of Microbiology, Hazara University, Mansehra, Pakistan
4. Health Research Institute, National Institute of Health, Research Centre, King Edward Medical University, Lahore, Pakistan

**Correspondence:** Dr. Shafiq ur Rehman, Associate Professor, Institute of Microbiology and Molecular Genetics, University of the Punjab, Lahore, Pakistan:

email: [shafiq.mmg@pu.edu.pk](mailto:shafiq.mmg@pu.edu.pk)

**Table S1: Antibiotic susceptibility results of Klebsiella pneumoniae isolates.**

**Abbreviations:** AMC; Amoxicillin-clavulanic acid, TZP; Piperacillin-tazobactam, FOX; Cefoxitin, CXM; Cefixime, CRO; Ceftriaxone, CFM; Cefuroxime, CAZ; Ceftazidime, SCF; Cefoperazone-sulbactam, FEP; Cefepime, AZT; Aztreonam, Ak; Amikacin, CN; Gentamycin, Do; Doxycycline, TET; Tetracycline, CIP, Ciprofloxacin, ENR; Enrofloxacin, PB; Polymyxin B, CT, Colistin, E; Erythromycin, NIT; Nitrofurantoin, C; Chloramphenicol, IPM, Imipenem, SXT; Trimethoprim-Sulfamethoxazole.

|  | | ***Klebsiella pneumoniae* strains antibiotic susceptibility testing results** | | | | | | | | | | | | | | | | | | | | | | | | | | | |
| --- | --- | --- | --- | --- | --- | --- | --- | --- | --- | --- | --- | --- | --- | --- | --- | --- | --- | --- | --- | --- | --- | --- | --- | --- | --- | --- | --- | --- | --- |
| **SR. NO** | **LAB ID** | | **16s rRNA NCBI Accession #** | **SOURCES** | **AMC** | **TZP** | **FOX** | **CXM** | **CRO** | **CFM** | **CAZ** | **SCF** | **FEP** | **AZT** | **AK** | **CN** | **DO** | **TET** | **CIP** | **ENR** | **NOR** | **PB** | **CT** | **E** | **NIT** | **C** | **IMP** | **SXT** |  |
| 1 | Kp-8890 | |  | Blood | R | S | R | R | R | R | R | R | S | S | S | S | S | R | R | R | R | S | S | R | S | S | R | R |  |
| 2 | Kp-9819 | |  | Urine | R | R | R | R | R | R | R | R | S | S | S | S | S | S | R | R | S | S | S | R | R | R | R | R |  |
| 3 | Kp-32 | |  | Sputum | R | S | R | R | R | R | R | R | S | S | S | S | S | S | S | S | S | S | S | R | S | S | R |  |  |
| 4 | Kp-36 | |  | Blood | R | R | R | R | R | R | R | R | R | S | S | S | R | R | R | R | R | S | S | R | S | S | R | R |  |
| 5 | Kp-37 | |  | Sputum | R | S | R | R | R | R | R | R | S | S | S | S | S | S | S | S | S | S | S | R | S | S | R |  |  |
| 6 | Kp-11 | |  | Urine | R | S | R | R | R | R | R | R | S | S | S | S | S | S | S | S | S | S | S | R | S | S | R |  |  |
| 7 | Kp-KPU | |  | Urine | R | R | R | R | R | R | R | R | R | R | R | R | R | R | R | R | R | S | S | R | S | S | R | R |  |
| 8 | Kp-8873 | |  | Wound swab | R | R | R | R | R | R | R | R | R | R | R | R | R | R | R | R | R | S | S | R | R | S | R | R |  |
| 9 | Kp-8668 | |  | Wound swab | R | R | R | R | R | R | R | R | R | R | R | R | R | R | R | R | R | S | S | R | R | R | R | S |  |
| 10 | Kp-25 | |  | Tracheal secretion | R | R | R | R | R | R | R | R | R | S | R | R | R | R | R | R | R | S | S | R | R | R | S | R |  |
| 11 | Kp-Eb2 | |  | Sputum | R | S | R | S | S | R | S | S | S | S | R | R | R | R | S | R | R | S | S | R | S | S | S | R |  |

**Table S2: Demonstrating the viable count after 6, 12 and 24- hour’s phage treatment for 1-4 days old biofilm with MOI-1 and 0.1 with respect to zero-time point**

| **Age of Biofilm** | **Phage Treatment Time (h)** | **Average CFU/mL after Treatment at MOI-1** | **Average CFU/mL after Treatment at MOI-0.1** | **Average CFU/mL for Untreated Bacterial Biofilm Growth Control** | **Average Log Reduction at MOI-1 with respect to Zero-Time Point** | **Average Log Reduction at MOI-0.1 with respect to Zero-Time Point** | **p-value MOI-1 vs. MOI-0.1** | **p-value MOI-1 vs.**  **Zero-Time point** | **p-value MOI-0.1 vs.**  **Zero-Time Point** |
| --- | --- | --- | --- | --- | --- | --- | --- | --- | --- |
| Day 1 | 0 | 2.7E+04 | 2.7E+04 | 2.7E+04 | - | - | - | - | - |
|  | 6 | 9.97E+03 | 1.41E+04 | 1.50E+05 | 0.429657 | 0.278916 | 0.6348 | 0.0479 | 0.0924 |
|  | 12 | 1.72E+03 | 8.35E+03 | 3.65E+05 | 1.193112 | 0.506656 | 0.5088 | <0.0001 | 0.0004 |
|  | 24 | 4.41E+02 | 4.79E+03 | 1.63E+06 | 1.783696 | 0.747437 | 0.0692 | 0.0004 | 0.0007 |
| Day 2 | 0 | 1.84E+06 | 1.84E+06 | 1.84E+06 | - | - | - | - | - |
|  | 6 | 8.60E+05 | 9.85E+05 | 1.13E+07 | 0.365444 | 0.306507 | 0.9933 | 0.0110 | 0.0115 |
|  | 12 | 2.60E+05 | 4.30E+05 | 5.00E+07 | 0.88497 | 0.666474 | 0.559 | 0.0037 | 0.0057 |
|  | 24 | 7.90E+04 | 3.95E+05 | 2.50E+08 | 1.402316 | 0.703346 | 0.5290 | 0.0137 | 0.0238 |
| Day 3 | 0 | 2.05E+09 | 2.05E+09 | 2.05E+09 | - | - | - | - | - |
|  | 6 | 1.04E+09 | 1.11E+09 | 3.55E+09 | 0.307249 | 0.278959 | 0.8926 | 0.0138 | 0.0168 |
|  | 12 | 3.60E+08 | 5.15E+08 | 5.30E+09 | 0.76798 | 0.612475 | 0.2624 | 0.0005 | 0.0006 |
|  | 24 | 2.95E+08 | 5.00E+08 | 3.50E+09 | 0.85446 | 0.625312 | 0.5505 | 0.0045 | 0.0064 |
| Day 4 | 0 | 2.50E+09 | 2.50E+09 | 2.50E+09 | - | - | - | - | - |
|  | 6 | 1.31E+09 | 1.51E+09 | 3.40E+09 | 0.244457 | 0.182463 | 0.8715 | 0.1155 | 0.1743 |
|  | 12 | 5.45E+08 | 6.81E+08 | 5.50E+09 | 0.625331 | 0.528581 | 0.7597 | 0.0437 | 0.0045 |
|  | 24 | 4.40E+08 | 6.54E+08 | 5.34E+09 | 0.718275 | 0.54615 | 0.5827 | 0.0038 | 0.0053 |

**Table S3: Demonstrating the viable count after 6, 12 and 24- hour’s phage treatment for 1-4 days old biofilm with MOI-1 and 0.1 with respect to Untreated Biofilm Growth Control**

| **Age of Biofilm** | **Phage Treatment Time (h)** | **Average CFU/mL after Treatment at MOI-1** | **Average CFU/mL after Treatment at MOI-0.1** | **Average CFU/mL for Untreated Bacterial Biofilm Growth Control** | **Average Log Reduction at MOI-1 with respect to** **Untreated control** | **Average Log Reduction at MOI-0.1 with respect to Untreated control** | **p-value MOI-1 vs. MOI-0.1** | **p-value MOI-1**  **vs.**  **Untreated control** | **p-value MOI-0.1 vs.**  **Untreated control** |
| --- | --- | --- | --- | --- | --- | --- | --- | --- | --- |
| Day-1 | 6 | 9.97E+03 | 1.41E+04 | 1.50E+05 | 1.177759 | 1.027017 | 0.9947 | 0.0654 | 0.0818 |
|  | 12 | 1.72E+03 | 8.35E+03 | 3.65E+05 | 2.32727 | 1.640814 | 0.9886 | 0.0881 | 0.0085 |
|  | 24 | 4.41E+02 | 4.79E+03 | 1.63E+06 | 3.567749 | 2.53149 | 0.9997 | 0.0084 | 0.0085 |
| Day-2 | 6 | 8.60E+05 | 9.85E+05 | 1.13E+07 | 1.116654 | 1.057716 | 0.9874 | 0.5383 | 0.0457 |
|  | 12 | 2.60E+05 | 4.30E+05 | 5.00E+07 | 2.283997 | 2.065502 | 0.9990 | 0.0025 | 0.0021 |
|  | 24 | 7.90E+04 | 3.95E+05 | 2.50E+08 | 3.500313 | 2.801343 | 0.7682 | 0.0014 | 0.0024 |
| Day-3 | 6 | 1.04E+09 | 1.11E+09 | 3.55E+09 | 0.533195 | 0.504905 | 0.9903 | 0.4836 | 0.4993 |
|  | 12 | 3.60E+08 | 5.15E+08 | 5.30E+09 | 1.167973 | 1.012469 | 0.8471 | 0.0321 | 0.0153 |
|  | 24 | 2.95E+08 | 5.00E+08 | 3.50E+09 | 1.074246 | 0.845098 | 0.6932 | 0.0429 | 0.0483 |
| Day-4 | 6 | 1.31E+09 | 1.51E+09 | 3.40E+09 | 0.414208 | 0.352214 | 0.9875 | 0.3419 | 0.5433 |
|  | 12 | 5.45E+08 | 6.81E+08 | 5.50E+09 | 1.003966 | 0.907216 | 0.9889 | 0.0458 | 0.0495 |
|  | 24 | 4.40E+08 | 6.54E+08 | 5.34E+09 | 1.084089 | 0.911964 | 0.8568 | 0.0431 | 0.0498 |

**Table S4: Statistical analysis of different treatment time-points at MOI-1 and 0.1 in comparison to zero-time point**

| **Age of Biofilm** | **Counts in comparison to Zero-Time point at MOI-1** | | | **Counts in comparison to Zero-Time point at MOI-0.1** | | |
| --- | --- | --- | --- | --- | --- | --- |
|  | **p value 6 vs. 12** | **p value 6 vs. 24** | **p value 12 vs. 24** | **p value 6 vs. 12** | **p value 6 vs. 24** | **p value 12 vs. 24** |
| Day 1 | 0.0018 | 0.0012 | 0.2349 | 0.0418 | 0.0210 | 0.7002 |
| Day 2 | 0.0049 | 0.0023 | 0.1214 | 0.0227 | 0.0202 | 0.9900 |
| Day 3 | 0.0547 | 0.0407 | 0.9792 | 0.0400 | 0.0374 | 0.9928 |
| Day 4 | 0.2603 | 0.2448 | 0.9998 | 0.6378 | 0.7134 | 0.829 |

**Table S5: Statistical analysis of different treatment time-points at MOI-1 and 0.1 with**

**respect to Untreated Biofilm Growth Control**

| **Age of Biofilm** | **Counts in comparison to Zero-Time point at MOI-1** | | | **MOI-0.1** | | |
| --- | --- | --- | --- | --- | --- | --- |
|  | **p value 6 vs. 12** | **p value 6 vs. 24** | **p value 12 vs. 24** | **p value 6 vs. 12** | **p value 6 vs. 24** | **p value 12 vs. 24** |
| Day 1 | 0.0018 | 0.0012 | 0.2349 | 0.0418 | 0.0210 | 0.7002 |
| Day 2 | 0.0049 | 0.0023 | 0.1214 | 0.0227 | 0.0202 | 0.9900 |
| Day 3 | 0.0472 | 0.0315 | 0.9407 | 0.0400 | 0.0374 | 0.9928 |
| Day 4 | 0.0341 | 0.0241 | 0.7990 | 0.6378 | 0.7134 | 0.829 |

**Table S6: Comprehensive table for JKP2 genome annotation. Assigned functions were based on the homology search by HHpred, HMMER, InterProscan, Cath, UniProtKB, NCBI blastp and RAST server. (Detailed table is present as table S2.2 in excel file)**

| **ORF** | **start** | **stop** | **strand** | **Gene Size** | **Assigned Function** |
| --- | --- | --- | --- | --- | --- |
| ORF1 | 720 | 1010 | + | 291 | hypothetical protein |
| ORF2 | 1041 | 1343 | + | 303 | terminase small subunit |
| ORF3 | 1343 | 3199 | + | 1857 | terminase large subunit |
| ORF4 | 3199 | 3573 | + | 375 | hypothetical protein |
| ORF5 | 3585 | 3767 | + | 183 | hypothetical protein |
| ORF6 | 3767 | 4171 | + | 405 | spanin |
| ORF7 | 4164 | 4415 | + | 252 | holin |
| ORF8 | 4399 | 4995 | + | 597 | Endolysin |
| ORF9 | 6502 | 6717 | + | 216 | hypothetical protein |
| ORF10 | 6796 | 7371 | + | 655 | hypothetical protein |
| ORF11 | 7869 | 8078 | + | 210 | hypothetical protein |
| ORF12 | 8071 | 8334 | + | 264 | hypothetical protein |
| ORF13 | 8347 | 8586 | + | 240 | hypothetical protein |
| ORF14 | 8589 | 8768 | + | 180 | hypothetical protein |
| ORF15 | 8765 | 8971 | + | 207 | hypothetical protein |
| ORF16 | 8959 | 9303 | + | 345 | hypothetical protein |
| ORF17 | 9281 | 11023 | + | 1743 | hypothetical protein |
| ORF18 | 11023 | 12069 | + | 1047 | peptidase |
| ORF19 | 12072 | 12518 | + | 447 | hypothetical protein |
| ORF20 | 12511 | 12657 | + | 147 | hypothetical protein |
| ORF21 | 12667 | 13452 | + | 786 | DNA primase |
| ORF22 | 13449 | 13643 | + | 195 | hypothetical protein |
| ORF23 | 13644 | 14924 | + | 1281 | Phage DNA helicase |
| ORF24 | 14976 | 15131 | + | 156 | hypothetical protein |
| ORF25 | 15274 | 17664 | + | 2391 | DNA-directed DNA polymerase |
| ORF26 | 17661 | 18209 | + | 549 | Nucleotidyltransferase |
| ORF27 | 18199 | 18417 | + | 219 | hypothetical protein |
| ORF28 | 18579 | 19559 | + | 981 | Phage phosphoesterase |
| ORF29 | 19822 | 20667 | + | 846 | large tegument protein |
| ORF30 | 20721 | 20975 | + | 255 | hypothetical protein |
| ORF31 | 20979 | 21353 | + | 375 | hypothetical protein |
| ORF32 | 21353 | 21514 | + | 162 | hypothetical protein |
| ORF33 | 21517 | 21714 | + | 198 | hypothetical protein |
| ORF34 | 21717 | 21878 | + | 162 | hypothetical protein |
| ORF35 | 21878 | 22384 | + | 507 | HNH endonuclease |
| ORF36 | 22359 | 23333 | + | 975 | 5'-3' exonuclease |
| ORF37 | 23290 | 23490 | + | 201 | hypothetical protein |
| ORF38 | 23484 | 23714 | + | 231 | Transcription elongation factor |
| ORF39 | 23921 | 24133 | + | 213 | DNA endonuclease VII |
| ORF40 | 24130 | 24591 | + | 462 | nucleotide kinase |
| ORF41 | 24733 | 27201 | + | 2469 | RNA polymerase |
| ORF42 | 27211 | 27549 | + | 339 | YspA-related SLOG family |
| ORF43 | 27573 | 28013 | + | 441 | hypothetical protein |
| ORF44 | 28010 | 28273 | + | 264 | hypothetical protein |
| ORF45 | 28283 | 29878 | + | 1596 | portal protein |
| ORF46 | 29893 | 30735 | + | 843 | scaffolding protein |
| ORF47 | 30761 | 31780 | + | 1020 | Major Capsid protein |
| ORF48 | 31792 | 31974 | + | 183 | capsid scaffolding protein |
| ORF49 | 32062 | 32622 | + | 561 | tail tubular protein A/exopolysaccharide depolymerase/type 3 adaptor protein |
| ORF50 | 32632 | 34992 | + | 2361 | tail tubular protein B/head closure protein |
| ORF51 | 34994 | 35581 | + | 588 | internal virion protein A |
| ORF52 | 35598 | 38282 | + | 2685 | internal virion protein B |
| ORF53 | 38333 | 42031 | + | 3699 | internal core protein |
| ORF54 | 42713 | 42129 | - | 585 | tail fiber protein |

**Table S7: Promotor sequences as detected by the PhagePromotor**

| **#** | **Strand** | **Positions** | **Promoter Sequence** | **Type** | **Scores** |
| --- | --- | --- | --- | --- | --- |
|  | + | (1009..1016) | GATAAATA | host | 0.512 |
|  | + | (2107..2136) | TGAAATGGAAGAGCGATATGGAAGTACACT | host | 0.661 |
|  | + | (2637..2666) | TTGATAAACTGATTGACCTGTGCGTAGAGT | host | 0.558 |
|  | + | (5964..5971) | GATAAATA | host | 0.736 |
|  | + | (6118..6146) | TTGACACCGCGAAGAACATAAGCTAGATT | host | 0.949 |
|  | + | (6187..6215) | TTGACAAGTTCCAATTCACTGAGTAACTT | host | 0.815 |
|  | + | (6241..6261) | CGGGGAGGCCCAGTACCTTGA | phage | 0.588 |
|  | + | (6722..6753) | AGCCTATAGCATCCTATGGGGTGCTATGTGAA | phage | 0.996 |
|  | + | (6971..6997) | TTGATATGGTGCTGCGTACGCTATTAC | host | 0.530 |
|  | + | (7601..7623) | AATCATGCCACAACAAAGGAGAG | phage | 0.521 |
|  | + | (7809..7840) | AGCCTATAGCGTCCTACGGGGCGCTATGTGAA | phage | 1.000 |
|  | + | (11045..11072) | TTGATATGCAGCTGCTCTTGCAGATACT | host | 0.533 |
|  | + | (12066..12091) | TTGATTATGTCTAAGATTTTTAAAGT | host | 0.550 |
|  | + | (21959..21987) | TTGAAAGCTCCTAAGTACAGCCATATTGT | host | 0.570 |
|  | + | (22381..22409) | TTGACTTGAGCGCCCTAGAGCCTCAAGAT | host | 0.670 |
|  | + | (37594..37619) | TTGACAAGAAGGGCAAATTCCAGGAT | host | 0.511 |
|  | + | (39483..39510) | TTGACAGTAATCCCGAGATTGCTAAACG | host | 0.546 |
|  | + | (40488..40519) | TTGCCAAGGAGATGGTTGACGGCATCTACAAT | host | 0.774 |
|  | + | (42967..42998) | TTGCCAGTAAATCCCTGTACCTGCATTATGAT | host | 0.900 |

**Table S8: Showing rho-independent terminators in JKP2 genome identified by ARNold web tool.**

| **#** | **Strand** | **Positions** | **Promoter Sequence** | **Scores** |
| --- | --- | --- | --- | --- |
|  | + | (660..706) | GGTCTGATTTATATCCCGCCCCACGGGGCGGGGTGTATTATTTGCTA | -11.90 |
|  | + | (730..767) | TAATAAGTAAAACGCTGGAAGCCAACGTCATCGCAGAT | -6.60 |
|  | + | (3907..3948) | CTTGAATAACAAGGTGGACTCCAGCACCACCAATAAAACCGA | -7.30 |
|  | + | (5501..5542) | ACTAGGCTATCCACTGGCTATCGCTAGTGCTTTACATTGTGC | -9.40 |
|  | + | (8966..9004) | CAGTAGACGAAACAGCACTGCTGTGCTGGAGGCTGCTGG | -8.80 |
|  | + | (12897..12935) | TAGCCAAGCGCCTGCGGAAACGCAGAGTTTTATTTACGG | -8.40 |
|  | + | (14009..14050) | GCTGGAAATGAAGCACCTACAGCGCAAGTACGGTGACGGCGC | -7.90 |
|  | + | (20678..20718) | TCCTAACTAATATGGCCCCGCCTAGGGGCCTTAGAGGAAGC | -13.80 |
|  | + | (20769..20811) | GCCGAAGCTAAACGCGCCGATGCCAAAGCACAGTTTAACGAGC | -5.70 |
|  | + | (25604..26673) | ATGGCATCAAAAAAGCGCTTAATAAGGCACAGGAAACCCCGTACCGTATTAATAAGCGCATACTGGAATT | NA |
|  | + | (31969..32020) | GAGTAATACGCGCCCCTGGTGCCTTCGGGTGCCAGGGGCTTTTTTTTTGTCC | -16.60 |
|  | + | (31978..32019) | GCGCCCCTGGTGCCTTCGGGTGCCAGGGGCTTTTTTTTTGTC | -4.30 |
|  | + | (35167..35201) | GTTTAACATCAAGCAGCAGAAGCTGCAGGCTCAGAGC | -8.70 |
|  | + | (39144..39189) | CGAGAAACAATACTGCCTCCGTGAACATGAAAGCACAGCACGTAGT | -7.30 |

**
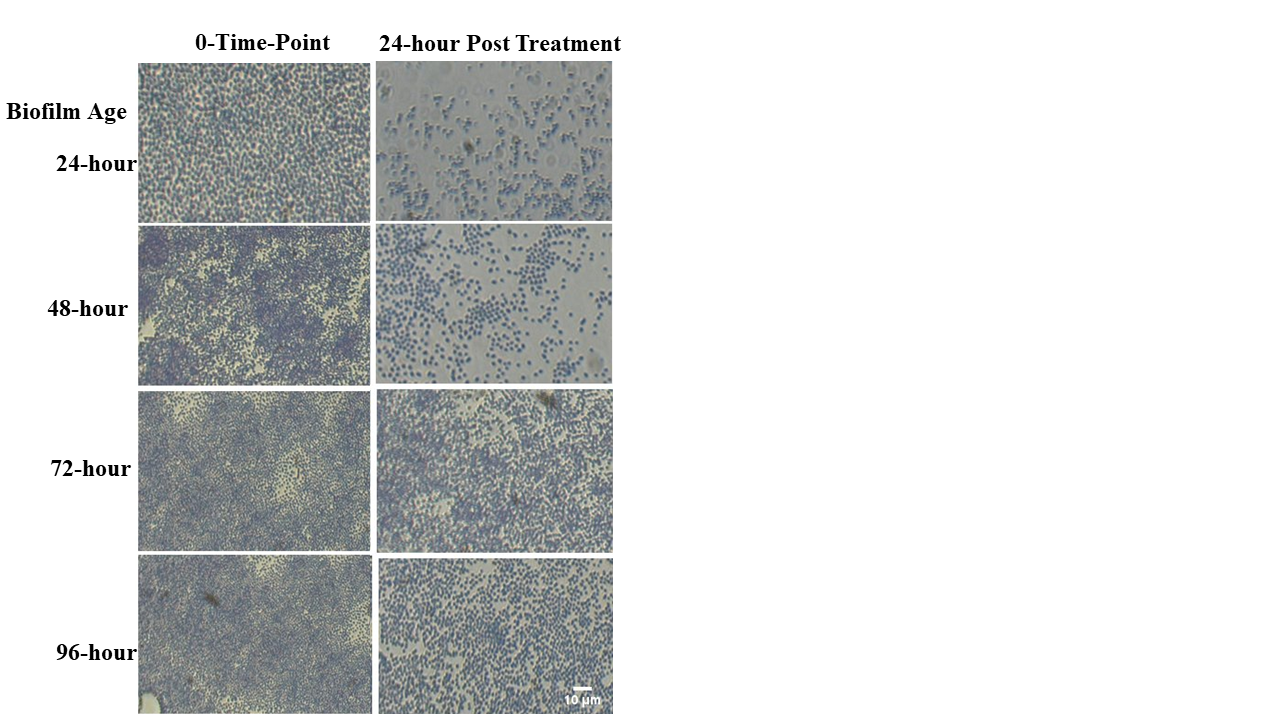
**

**Figure S1: CV stained biofilm of 24, 48, 72 and 96-hours age with changes in biofilm population after phage treatment for 24-hours. Image were taken after CV staining of microtiter plate wells by using inverted microscope at 20X magnification.**

**Figure S2: Repeat Masker output snapshot showing sequence of simple repeats with their location in the genome**.


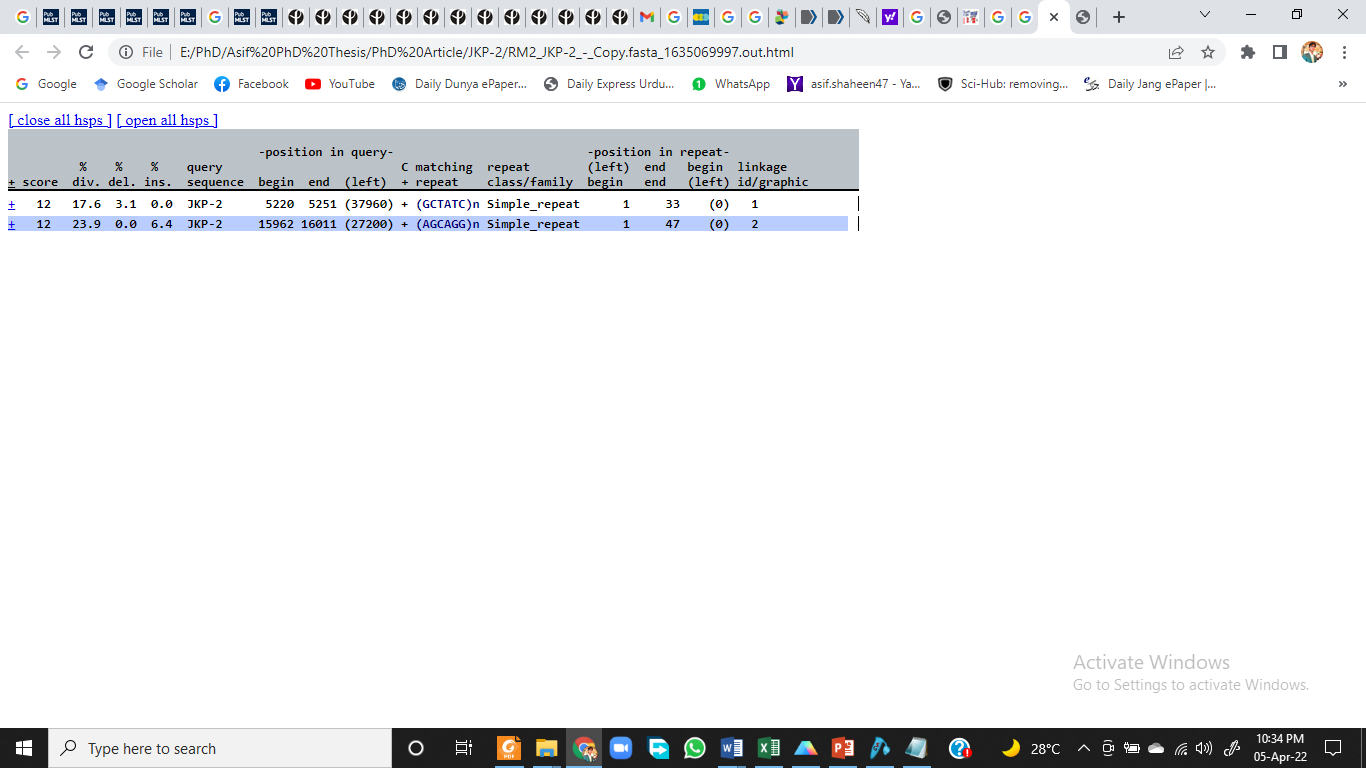


**Figure S3: Tandem Repeat Finder output snapshot showing sequence of tandem repeats with their location in the genome**.


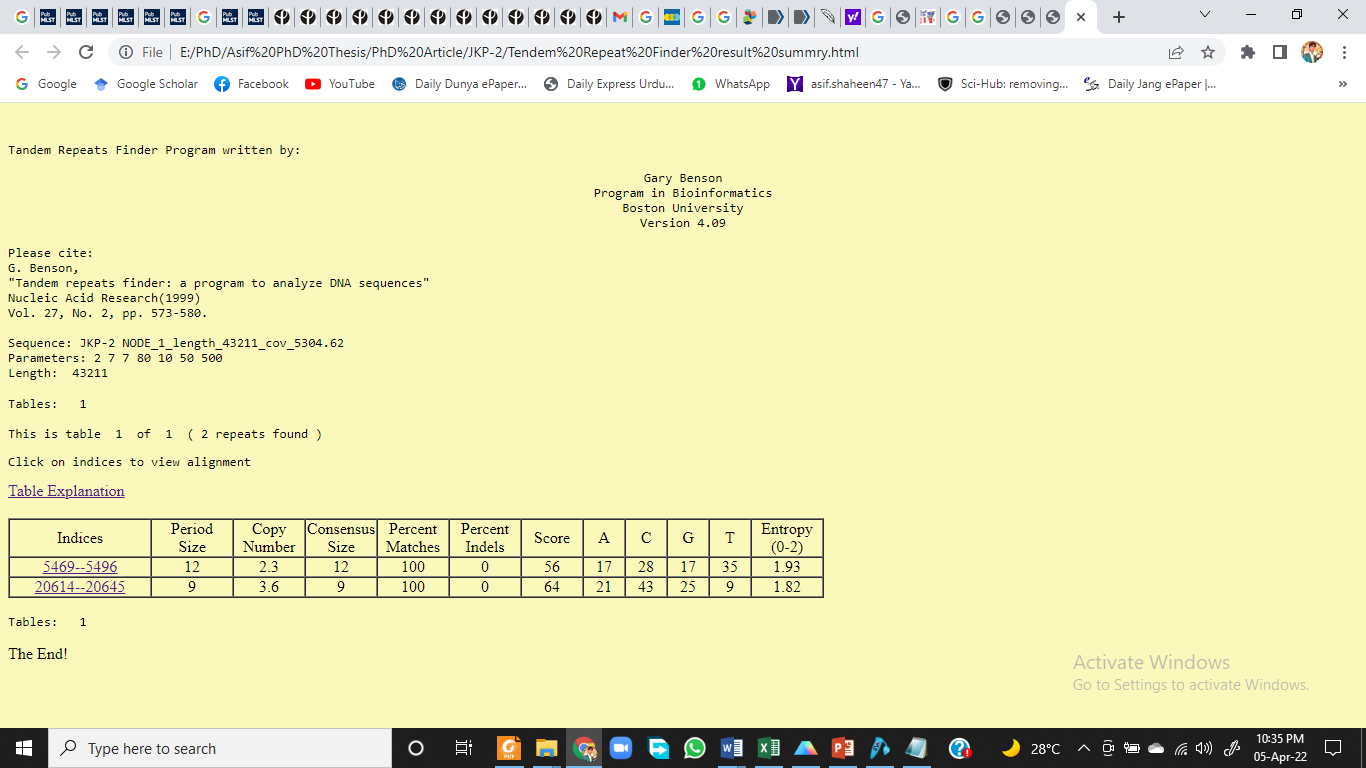


**Figure S4: Output file of effective DB for type-III secretion system identification**


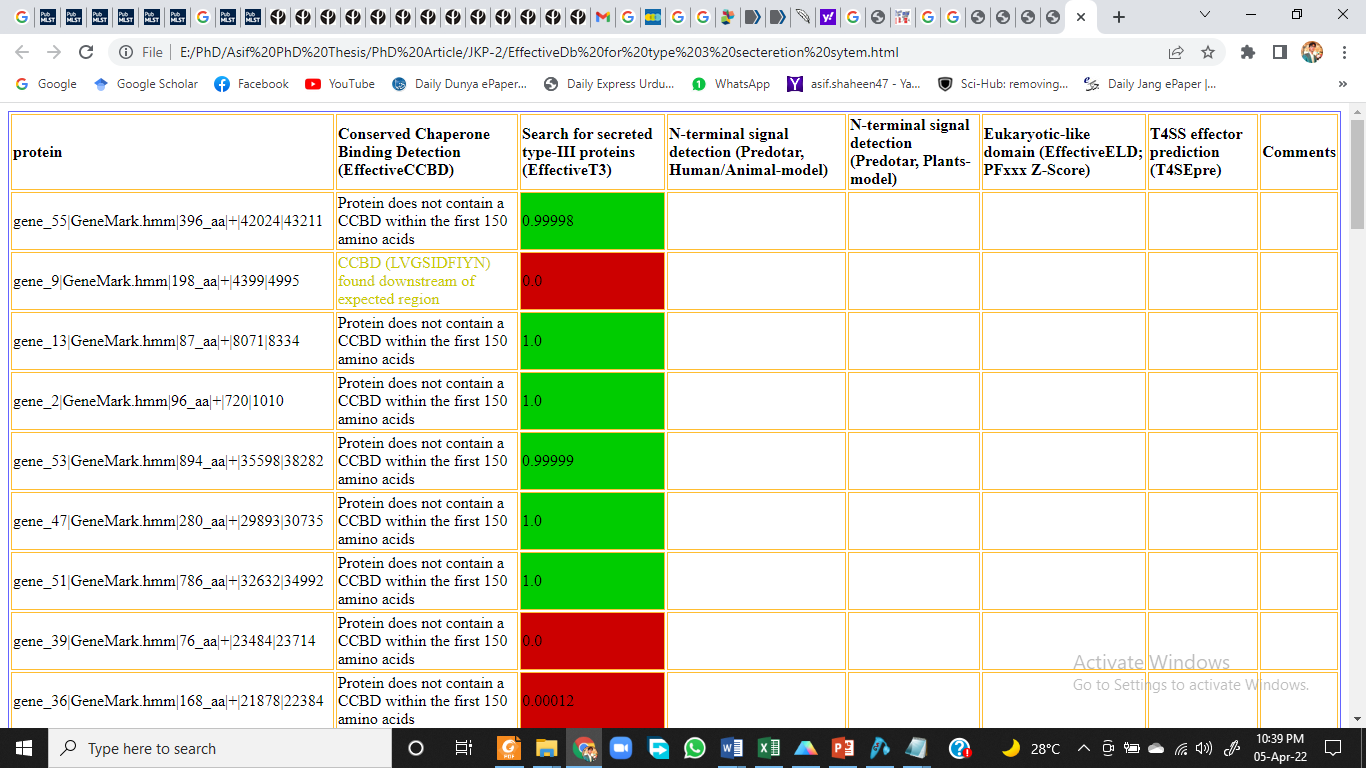


**Figure S5: GC-skew plot for JKP2**


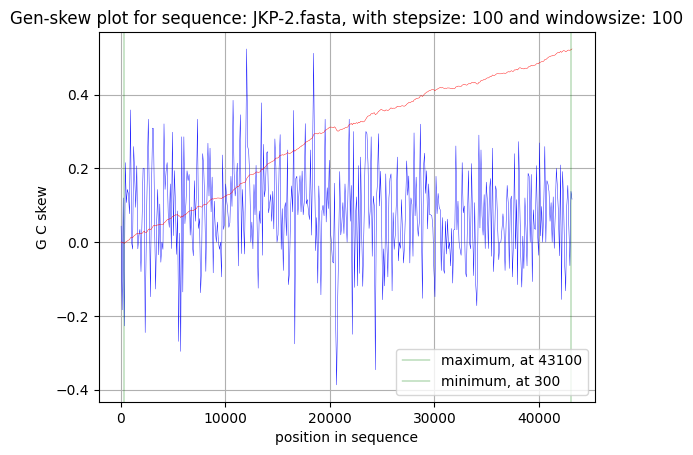


**Figure S6: VipTree generated proteomic tree of JKP2. It mad cluster with Autographiviridae family.**


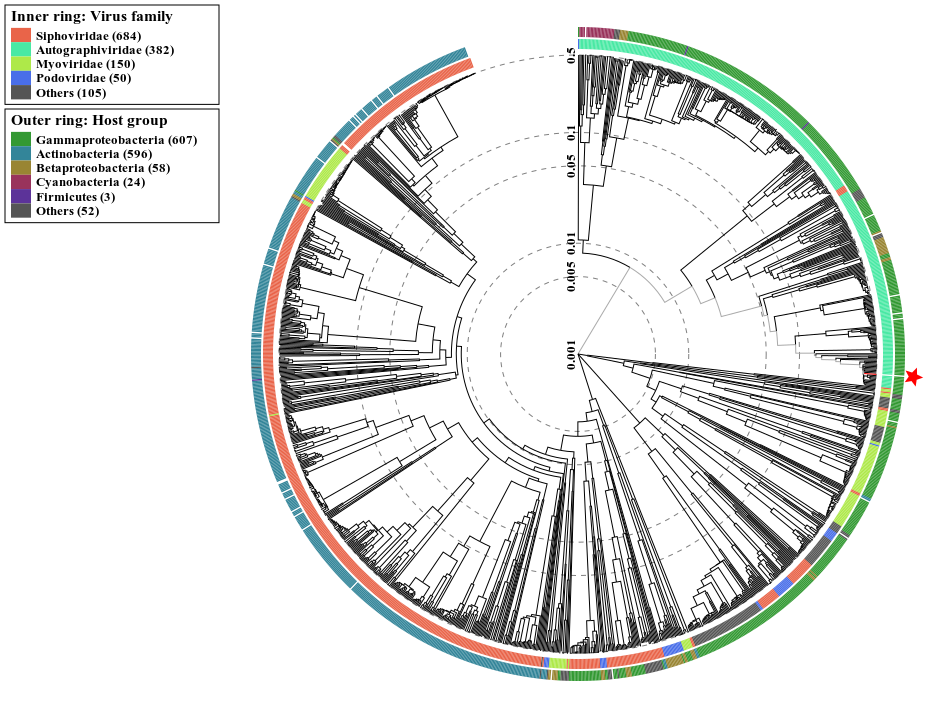


**Figure S7: Virfam generated detection and localization of** head neck tail module **in the genome of JKP2.**


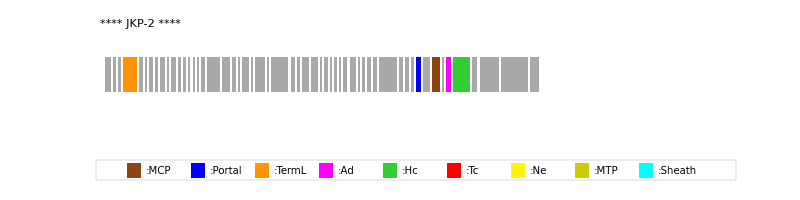

Supplement: Supplementary file 1 [file mmc1.docx]
